# Safety and efficacy of a freeze-dried trivalent antivenom for snakebites in the Brazilian Amazon: An open randomized controlled phase IIb clinical trial

**DOI:** 10.1371/journal.pntd.0006068

**Published:** 2017-11-27

**Authors:** Iran Mendonça-da-Silva, Antônio Magela Tavares, Jacqueline Sachett, José Felipe Sardinha, Lilian Zaparolli, Maria Fátima Gomes Santos, Marcus Lacerda, Wuelton Marcelo Monteiro

**Affiliations:** 1 Escola Superior de Saúde, Universidade do Estado do Amazonas, Manaus, Amazonas, Brazil; 2 Departamento de Ensino e Pesquisa, Fundação de Medicina Tropical Dr. Heitor Vieira Dourado, Manaus, Amazonas, Brazil; 3 Instituto de Biologia do Exército, Rio de Janeiro, Rio de Janeiro, Brazil; 4 Laboratório Químico e Farmacêutico do Exército, Rio de Janeiro, Rio de Janeiro, Brazil; 5 Instituto Leônidas & Maria Deane, Fundação Oswaldo Cruz, Manaus, Amazonas, Brazil; Instituto de Biomedicina de Valencia, SPAIN

## Abstract

**Background:**

In tropical areas, a major concern regarding snakebites treatment effectiveness relates to the failure in liquid antivenom (AV) distribution due to the lack of an adequate cold chain in remote areas. To minimize this problem, freeze-drying has been suggested to improve AV stability.

**Methods and findings:**

This study compares the safety and efficacy of a freeze-dried trivalent antivenom (FDTAV) and the standard liquid AV provided by the Brazilian Ministry of Health (SLAV) to treat *Bothrops*, *Lachesis* and *Crotalus* snakebites. This was a prospective, randomized, open, phase IIb trial, carried out from June 2005 to May 2008 in the Brazilian Amazon. Primary efficacy endpoints were the suppression of clinical manifestations and return of hemostasis and renal function markers to normal ranges within the first 24 hours of follow-up. Primary safety endpoint was the presence of early adverse reactions (EAR) in the first 24 hours after treatment. FDTAV thermal stability was determined by estimating AV potency over one year at 56°C. Of the patients recruited, 65 and 51 were assigned to FDTAV and SLAV groups, respectively. Only mild EARs were reported, and they were not different between groups. There were no differences in fibrinogen (p = 0.911) and clotting time (p = 0.982) recovery between FDTAV and SLAV treated groups for *Bothrops* snakebites. For *Lachesis* and *Crotalus* snakebites, coagulation parameters and creatine phosphokinase presented normal values 24 hours after AV therapy for both antivenoms.

**Conclusions/Significance:**

Since promising results were observed for efficacy, safety and thermal stability, our results indicate that FDTAV is suitable for a larger phase III trial.

**Trial registration:**

ISRCTNregistry: ISRCTN12845255; DOI: 10.1186/ISRCTN12845255 (http://www.isrctn.com/ISRCTN12845255).

## Author summary

Antivenoms (AV) are included in the WHO List of Essential Medicines, being the only treatment available for snakebites envenomings. In Brazil, five types of liquid snake AVs are distributed by the Ministry of Health for national use free of charge to patients. In remote areas, lack of an adequate cold chain impairs AV distribution to health facilities resulting in delay in patient care and, ultimately, in higher complication and case fatality rate. To minimize this problem, a freeze-drying process has been suggested to improve the stability of AVs, but freeze-dried AVs efficacy and safety evidence obtained from clinical trials is still very limited. Freeze-drying is a process by which water is removed from a sample without the need to apply heat. Benefits of lyophilization are: a) Samples are processed in aseptic conditions; b) Process does not use heat, ensuring formulation stability; c) Increases shelf life; d) Samples can be stored at room temperature for a long time; and e) Reduces weight and volume of samples, which is ideal for distribution. In this study, a freeze-dryed formulation is presented as a good alternative for a more stable trivalent antivenom in regions of the Amazon where high temperatures are common and the cold chain is poor. Our results suggest that such a product is adequate for a phase III trial.

## Introduction

Snakebites are a serious public health problem in tropical countries, with higher morbidity and case fatality rates in poor, underdeveloped, rural and remote rainforest areas. At least 421,000 envenomings and 20,000 deaths occur each year due to snakebites globally, but these figures may be as high as 1,841,000 envenomings and 94,000 deaths [1]. Additionally, 400,000 amputations and other severe health consequences such as infection, tetanus, scarring, contractures, and psychological sequelae have been recorded [2]. In Brazil, from 2000 to 2015 a total of 416,109 snakebites were recorded by the Brazilian official surveillance system, with 26,000 cases on average per year. Snakebites incidence is higher in the Brazilian Amazon states, with a rate of 55.4 cases/100,000 inhabitants in 2015, and considered an occupational health problem of rural and riverine populations [3]. However, the true burden of snakebites is probably higher and difficult to estimate since only a few countries have a reliable system for epidemiological surveillance of these events [4]. In the Brazilian Amazon, the case fatality rate has been estimated to be 0.6% and associated with older age and delayed medical assistance [5].

The *Bothrops* genus is responsible for 80–90% of snakebites in the Brazilian Amazon [5,6,7]. In this region, *Bothrops* envenoming results in pain, swelling, regional lymphadenopathy, ecchymosis, blistering, and necrosis as the most common local manifestations [6,8,9]. Systemic bleeding and acute renal failure are common systemic complications after *Bothrops* envenomings [6,9]. Clinical manifestations at the *Bothrops* and *Lachesis* bite sites are similar, with an intense tissue damage evidenced by pain, edema, blisters, bleeding, and ecchymosis [10]. *Bothrops* and *Lachesis* venoms are characterized by three main pathophysiological activities: coagulant, hemorrhagic, and proteolytic or acute inflammatory effects [11–14]. Signs and symptoms of vagal stimulation, such as dizziness, blurred vision, diarrhea, abdominal cramps, sinus bradycardia, severe hypotension and shock, may occur after *Lachesis* bites [10,15]. *Crotalus* venom has neurotoxic, myotoxic and coagulant activities and its systemic manifestations include drowsiness, ptosis, ophthalmoplegia, sagging face muscles, blurred vision, diplopia, myalgia, arthralgia and myoglobinuria, with mild clinical manifestations at the bite site generally [12].

Currently, antivenom (AV) immunoglobulins, included in the WHO List of Essential Medicines, are the only treatment available for snakebites envenomings [16]. In Brazil, the Ministry of Health implemented the National Program for Snakebites Control in 1986 [17]. Since then, AV production has been carried out by national laboratories and distributed by the Ministry of Health for national use free of charge to patients. Five types of snake liquid AVs are currently available: *Bothrops* AV, *Crotalus* AV, *Bothrops-Crotalus* AV, *Bothrops-Lachesis* AV, and *Micrurus* AV [4]. In the Amazon and other tropical areas, a major concern regarding snakebites treatment effectiveness relates to the impossibility of wide and timely liquid AV distribution. The lack of an adequate cold chain impairs AV distribution to health facilities available in remote areas and may result in delayed patient care and, ultimately, in higher complications and case fatality rates [5]. In additon, inadequate storage and transportation may result in loss of material [4]. To minimize this problem, it has been suggested freeze-drying to improve the stability of AV immunoglobulins. This additional step and addition of different stabilizers lead to AVs with a higher stability compared to liquid formulations, especially in tropical regions where high temperatures could affect the activity of immunoglobulins [18–20]. Physicochemical characterization studies of commercial freeze-dried AVs from India, Mexico, Thailand and Costa Rica showing good quality standards are available in the literature [18,20]. However, knowledge about freeze-dried AVs efficacy and safety obtained from clinical trials is still very limited.

This study was performed to compare the safety and efficacy of a freeze-dried trivalent antivenom with those of the available AV provided by the Brazilian Ministry of Health (MoH), to treat *Bothrops*, *Lachesis* and *Crotalus* snakebites in the Brazilian Amazon.

## Materials and methods

### Study design and participants

This was a prospective, randomized, open, phase IIb trial, carried out from June 2005 to May 2008. *Bothrops* snakebites from the *Tropical Medicine Foundation Dr*. *Heitor Vieira Dourado* (FMT-HVD), Manaus, Amazonas state, Brazil were included. *Lachesis* snakebites included were from the local hospitals at Borba (Amazonas state, Brazil), Normandia and Pacaraima (Roraima state, Brazil). *Crotalus* snakebites were included from the local hospitals of Boa Vista and Cantá (Roraima state, Brazil). Eligible patients were male and female subjects aged between 12 and 70 years old. *Bothrops*, *Lachesis* and *Crotalus* snakebites were diagnosed using clinical, epidemiological and laboratorial evaluations. Exclusion criteria were pregnancy or breastfeeding, previous hematological disorders, known immunodeficiencies (HIV, malignancies, chemotherapy or other immunosuppressive treatments), previous treatment with snake AVs and history of any moderate/severe allergic reactions. Moreover, patients presenting with severe snake envenomings, defined for *Bothrops* and *Lachesis* as life-threatening snakebites with severe bleeding, hypotension, shock and acute renal failure, and for *Crotalus* as intense rhabdomyolisis and severe acute renal failure [21], were not included.

### Patients’ selection, interventions and baseline clinical characterization

After snakebite envenoming diagnosis, patients that fulfilled all inclusion criteria were randomly assigned with allocation ratio 1:1 to one of the following groups:

1. **Group A**: Freeze-dried trivalent antivenom (FDTAV), produced under GMP conditions by *Butantan Institute* (São Paulo, Brazil) in partnership with *Instituto de Biologia do Exército* (Rio de Janeiro, Brazil). In this study, freeze-drying was performed at the Butantan Institute. Briefly, 20 mL vials were filled with 5 mL of each formulation and loaded on a freeze-dryer Benchmark 1100 (Virtis, USA). The samples were frozen at -40°C and annealed at -10°C for 4 h. The primary drying was conducted at -20°C for 64 h, and the secondary drying at 30°C for 4 h and 200 mTorr.
2. **Group B**: Available *Bothrops*, *Bothrops-Lachesis* and *Bothrops-Crotalus* AVs provided by the MoH (SLAV). In Brazil, snake AV production is standardized and all the AV production from the three national laboratories (*Butantan Institute*, *Ezequiel Dias Foundation* and *Vital Brazil Institute*) is acquired by the MoH for national distribution free of charge.

A randomization list was computer-generated. When a patient was considered to meet the inclusion criteria and had given her/his informed consent, he/shewas formally recruited and a unique ID number was allocated in the Case Report Form (CRF). After admission, a CRF was filled with the patient's unique ID number, gender, area of occurrence of the snakebite (rural or urban), age (in years), ethnicity, education (in years), anatomical region of the bite, and time from bite to medical assistance (in hours). Clinical examination included the observation of local and systemic manifestations. For *Bothrops* snakebites, laboratorial characterization included clotting time, erythrocyte sedimentation rate, International Normalized Ratio (INR), hemoglobin, leucocyte and platelet counts and plasma levels of fibrinogen, creatinine, urea, lactate dehydrogenase, aspartate transaminase, alanine transaminase and creatine phosphokinase in the plasma. For *Lachesis* and *Crotalus* snakebites, laboratorial characterization included clotting time, INR and plasma levels of fibrinogen, creatinine, urea, and activities of aspartate transaminase, alanine transaminase and creatine phosphokinase in the plasma.

Twenty minutes after pre-medication with IV hydrocortisone (500 mg), IV cimetidine (300 mg) and oral dexchlorpheniramine (5 mg) (standardized according to local guidelines), AV therapy was given to all patients from both groups in a dosage corresponding to mild or moderate envenomation. For detailed information about the allocation groups and interventions, see Table 1. Before administration, each FDTAV vial was reconstituted in 20 mL of sterile saline solution (0.9%) with a further dilution in 100 mL before administration. Each SLAV vial was diluted in 100 mL of saline solution 0.9%. Antivenom administration time ranged from 30 to 45 minutes.

**Table 1:** Description of the study allocation groups.

Study group	Intervention	Description of the interventional product	Venoms used for production	Dosage
**Group A**	Freeze-dried trivalent antivenom (AV) (FDTAV)	Each vial of FDTAV contains heterologous horse F(ab’)2, neutralizing at least 100 mg, 60 mg and 30 mg of the reference venoms of *Bothrops jararaca*, *Lachesis muta* and *Crotalus durissus terrificus*, respectively, in mice, sucrose (1 g), NaCl (0.17 g) and phenol (35 mg maximum). Each vial is accompanied by an ampoule containing 20 mL of sterile saline solution (0.9%)	*Bothrops* genus (*B*. *jararaca* 50.0%, *B*. *alternatus* 12.5%, *B*. *jararacuçu* 12.5%, *B*. *moojeni* 12.5% and *B*. *neuweidi* 12.5%), *Lachesis* genus (*L*. *muta* 100.0%), *Crotalus* genus (*C*. *durissus terrificus* 50.0% and *C*. *d*. *collilineatus* 50.0%)	*Bothrops* bites: 2 vials to mild cases and 4 vials to moderate cases *Lachesis* bites: 5 vials to non-severe cases *Crotalus* bites: 2,5 vials to mild cases and 5 vials to moderate cases
**Group B** [1]				
*Bothrops* bites	*Bothrops* AV provided by the MoH (SLAV)	Each vial contains heterologous horse F(ab’)2, neutralizing at least 50 mg of the reference venom of *Bothrops jararaca* in mice, phenol (35 mg maximum) and physiological solution 0.85% q.s. 10 mL	*Bothrops jararaca* (50.0%), *B*. *alternatus* (12.5%), *B*. *jararacuçu* (12.5%), *B*. *moojeni* (12.5%) and *B*. *neuweidi* (12.5%)	4 vials to mild cases and 8 vials to moderate cases
*Lachesis* bites	*Bothrops-Lachesis* AV provided by the MoH (BLMoHA)	Each vial contains heterologous horse F(ab’)2, neutralizing at least 50 mg and 30 mg of the reference venoms of *Bothrops jararaca* and *Lachesis muta*, respectively, in mice, phenol (35 mg maximum) and physiological solution 0.85% q.s. 10 mL	*Bothrops* genus (*B*. *jararaca* 50.0%, *B*. *alternatus* 12.5%, *B*. *jararacuçu* 12.5%, *B*. *moojeni* 12.5% and *B*. *neuweidi* 12.5%), *Lachesis* genus (*L*. *muta* 100.0%)	10 vials to non-severe cases
*Crotalus* bites	*Bothrops-Crotalus* AV provided by the MoH (BCMoHA)	Each vial contains heterologous horse F(ab’)2, neutralizing at least 50 mg and 15 mg of the reference venoms of *Bothrops jararaca* and *Crotalus durissus terrificus*, respectively, in mice, phenol (35 mg maximum) and physiological solution 0.85% q.s. 10 mL	*Bothrops* genus (*B*. *jararaca* 50.0%, *B*. *alternatus* 12.5%, *B*. *jararacuçu* 12.5%, *B*. *moojeni* 12.5% and *B*. *neuweidi* 12.5%), *Crotalus* genus (*C*. *durissus terrificus* 50.0% and *C*. *d*. *collilineatus* 50.0%)	5 vials to mild cases and 10 vials to moderate cases

^[1]^ Brazilian Ministry of Health (2001). Manual de diagnóstico e tratamento de acidentes por animais peçonhentos. Brasília: Brazilian Ministry of Health. 120 p.

Intravenous paracetamol was given on demand for pain. The bitten limb was nursed in the most comfortable position, blisters were aspirated, necrotic tissue was surgically debrided, abscesses were drained, and antibiotic treatment was given accordingly.

### Patients’ follow-up

After AV therapy, patients were admitted to the hospital ward for close monitoring during 24 hours. The same laboratorial tests referred above were repeated 4 hours (H4), 12 hours (H12) and 24 hours (H24) after AV therapy. Patients were asked to attend the hospital seven (D7) and fifteen days (D15) after discharge. At follow-up visits, clinical examination was carried out and the above mentioned laboratorial tests were performed in order to investigate clinical evolution of the envenomations and occurrence of late adverse reactions to AV therapy. If the patient did not present for the follow-up visits, the investigator planned a domiciliary visit the following day. Patients who did not present to hospital visits and were not found at domiciliary visits were considered lost to follow-up.

### Safety endpoints

Primary safety outcome was defined as the presence of signs and symptoms of early adverse reactions of AV therapy represented by urticaria, asthma-like crisis, laryngeal edema and shock, shortly after infusion until the first 24 hours after treatment. Early reactions were treated with intramuscular or subcutaneous adrenaline (1:1000, 0.3–0.5 mL), and repeated after 10 minutes if needed. After the symptoms of reaction had subsided, AV therapy was restarted.

Secondary safety outcome was defined as the presence of late adverse events, namely fever, urticarial, arthralgia, adenomegaly, neurological and renal complications, until D15. After D15, patients were advised to return to the hospital or to contact the study physician by phone in case of any signs of complications. In case of late reactions, treatment consisted of analgesics, anti-histamines and corticotherapy.

### Efficacy endpoints

For *Bothrops* and *Lachesis* snakebites, primary efficacy outcome was defined as the recovery of hemostasis parameters to normal ranges within the first 24 hours of follow-up. For *Crotalus* snakebites, primary efficacy outcome was defined as the suppression of coagulopathy and improvement of creatine phosphokinase activity within the first 24 hours of follow-up. Secondarily, suppression of neurological manifestations and recovery of other laboratorial parameters to normal ranges within the first 24 hours of follow-up was estimated in both groups. A second dose of four vials of AV was planned to be given if the patient’s blood remained totally incoagulable 12 hours after the initial dose, or in case of coagulopathy recurrence, again following the Brazilian Ministry of Health’s guidelines [21]. *Lachesis* and *Crotalus* patients were recruited in remote countryside areas, unfortunately with no availability to some laboratorial analysis used as secondary endpoints.

### Blinding

This was an open label trial that exclusively relied on laboratorial values for the assessment of the primary efficacy outcome, therefore blinding of laboratory staff was ensured. The laboratory personnel performing the analyses had no direct contact with the investigators and no information regarding the drug administered to the patient.

### Reconstitution test

Before administration, AV dissolution in 20 mL of sterile saline solution (0.9%) was observed visually as the FDTAV vials were gently agitated by hand for one minute. Successful reconstitution was considered when the dry AV was dissolved into a homogeneous solution.

### Quality control and stability

Samples of the FDTAV were sent to the *Instituto Nacional de Controle de Qualidade em Saúde* (INCQS), Fundação Oswaldo Cruz, Rio de Janeiro, Brazil, for quality control and stability evaluation. Total protein concentration and proportion of immunoglobulins, nonspecific toxicity, pyrogen levels, microbiological parameters and phenol concentrations were evaluated using standard methods. Anti-*Bothrops*, *Lachesis* and *Crotalus* potencies (mg/mL) were estimated by calculating the volume of FDTAV that neutralizes 1 mg of venom in mice. The FDTAV thermal stability was determined by estimating AV potency kept 15 days, one month, three months and one year at 56°C, compared to baseline potency. Methods employed for estimating AV potency [22] are presented in S1 File. Turbidity and presence of aggregates were observed by visual examination for quality control over time in parallel with potency and before AV administration.

### Statistical methods

Patients were distributed in two groups according the type of AV received. Baseline epidemiological, laboratorial and clinical characteristics of patients bitten by *Bothrops* were compared by Fisher test or chi-square test, using a 5% significance level. Student's t-test was used for comparison of means. For *Lachesis* and *Crotalus* patients, only a descriptive analysis was conducted because of the small number of cases. The primary efficacy analysis was done on all randomly selected patients finishing the follow-up (per protocol population). The primary efficacy endpoint for *Bothrops* bites, coagulopathy-free efficacy at 48 hours, was analysed using Kaplan-Meier estimates. A two-sided log-rank test was done over the time period using a 5% significance level. All other efficacy endpoints were analyzed with descriptive statistics. Primary safety endpoint frequencies between intervention and control groups were tested by Fisher test or chi-square test, using a 5% significance level, pooling all types of snakebites. Where zeros caused problems with computation of p values, 0.5 was added to all cells [23,24]. Statistical analyses were performed using the STATA statistical package version 13 (Stata Corp. 2013).

### Ethics

Eligible patients were asked to meet the study investigator, who gave detailed explanation of the study protocol according to the patient information sheet. Written consent was requested from the patient or from their parents/guardians for minors. The study protocol was approved by the Research Ethics Committee of the *Instituto de Biologia do Exército* (IBEx), Rio de Janeiro, Brazil, and by the National Ethical Committee (approval number 001-03/2003).

## Results

### Patients inclusion

**Fig 1:**
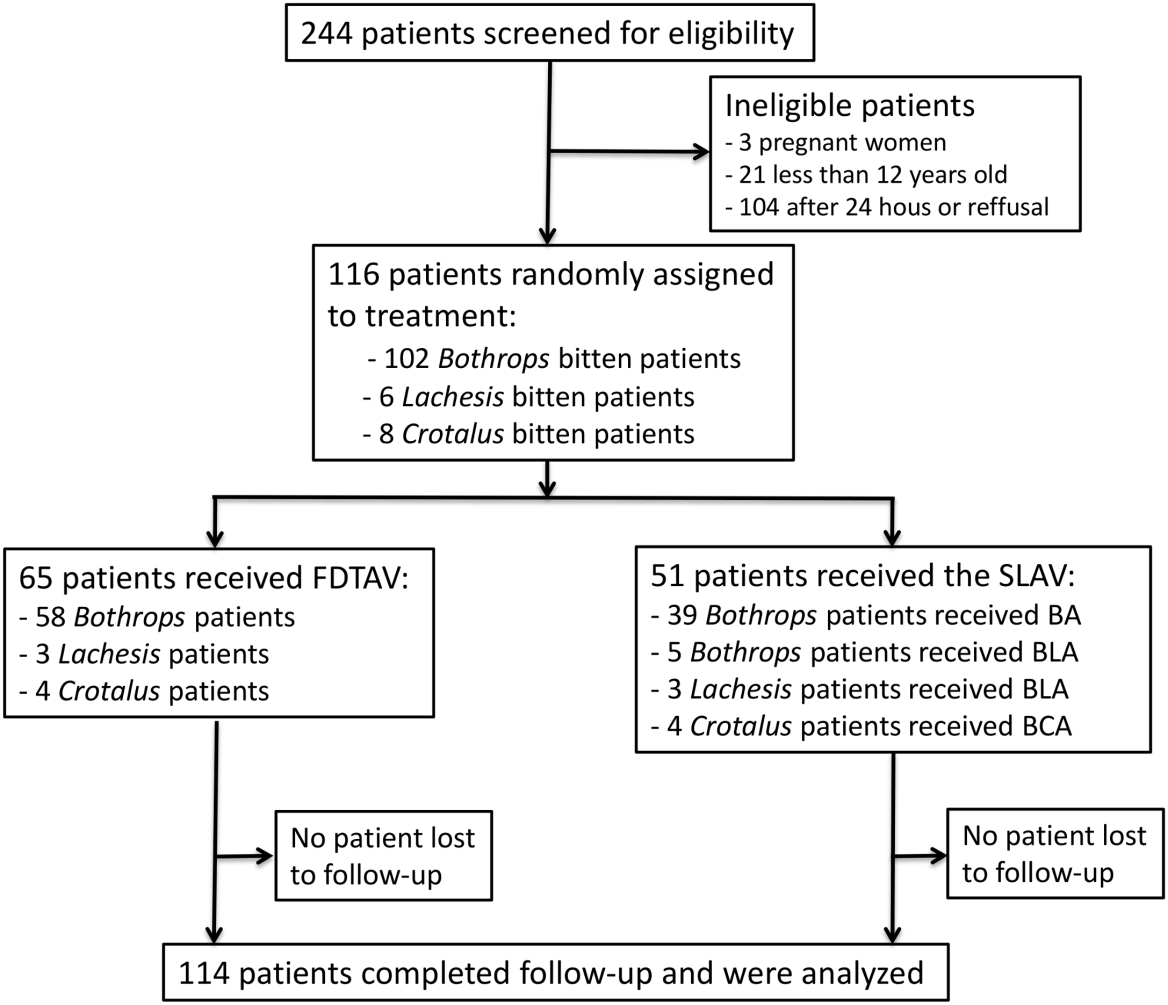
Flow chart of inclusion in the clinical trial.

The assessment and enrollment of ptients in this study is shown in a flow diagram (Fig 1). Briefly, out of 244 patients initially screened, 116 were eligible for inclusion: 102 cases of *Bothrops*, 6 cases of *Lachesis* and 8 cases of *Crotalus* snakebites. Of the patients recruited, 65 were assigned to Group A (FDTAV) (58 *Bothrops*, three *Lachesis* and four *Crotalus* bites) and 51 to the Group B (SLAV) (44 *Bothrops*, three *Lachesis* and four *Crotalus* bites). No patient was lost to follow-up.

### Baseline characterization

*Bothrops* snakebites were more frequent in males (88.2%), and mostly occurred in rural areas (68.6%). The most affected age group was the 16–30 years old (43.1%) followed by the 31–60 years old (42.2%). Admixed ethnicity was recorded for 95.1% of the included patients. A total of 50.0% of the patients presented 1–4 years of education. The most affected anatomical sites were the lower limbs (93.1%). Time elapsed from bite to medical assistance was higher than 6 hours in 34.3% of the cases. *Lachesis* and *Crotalus* bites showed the same general epidemiological characteristics (Table 2). Baseline laboratorial characteristics were similar in both groups (Table 3 and S2 File). The most frequent manifestations observed at admission in *Bothrops* snakebites were pain (96.1%), edema (96.1%) and local bleeding (30.4%). The most frequent systemic manifestations were nausea (11.8%), acute renal failure (6.9%) and bleeding (6.9%). Vagal manifestations, such as vomiting, bradycardia, hypotension, abdominal pain and blurred vision, were observed in *Lachesis* bites. Myalgia and neurological manifestations, namely palpebral ptosis, diplopia, ophtalmoplegia and dizziness, were recorded for *Crotalus* bite cases (Table 4). There were no deaths or permanent sequelae in the study.

**Table 2:** Epidemiological characteristics of the patients and comparison between experimental groups.

Variable	*Bothrops* bites (number, %)				*Lachesis* bites (number, %)			*Crotalus* bites (number, %)		
	Total	Group A	Group B	p	Total	Group A	Group B	Total	Group A	Group B
**Sex**										
Male	90 (88.2)	53 (91.4)	37 (84.0)	0.258	5 (83.3)	3 (100.0)	2 (66.7)	7 (87.5)	3 (75.0)	4 (100.0)
Female	12 (11.8)	5 (8.6)	7 (16.0)		1 (16.7)	0 (0.0)	1 (33.3)	1 (12.5)	1 (25.0)	0 (0.0)
**Area of occurrence**										
Rural	70 (68.6)	42 (72.4)	28 (63.6)	0.344	6 (100.0)	3 (100.0)	3 (100.0)	4 (50.0)	3 (75.0)	1 (25.0)
Urban/Periurban	32 (31.4)	16 (27.6)	16 (36.4)		0 (0.0)	0 (0.0)	0 (0.0)	4 (50.0)	1 (25.0)	3 (75.0)
**Age group (in years)**										
0–15	12 (11.8)	4 (6.9)	8 (18.2)	1	2 (33.3)	1 (33.3)	1 (33.3)	0 (0.0)	0 (0.0)	0 (0.0)
16–30	44 (43.1)	24 (41.4)	20 (45.4)	0.193	3 (50.0)	1 (33.3)	2 (66.7)	5 (62.5)	3 (75.0)	2 (50.0)
31–60	43 (42.2)	28 (48.3)	15 (34.1)	0.051	0 (0.0)	0 (0.0)	0 (0.0)	2 (25.0)	0 (0.0)	2 (50.0)
≥61	3 (2.9)	2 (3.4)	1 (2.3)	0.292	1 (16.7)	1 (33.3)	0 (0.0)	1 (12.5)	1 (25.0)	0 (0.0)
**Ethnicity**										
Admixed	97 (95.1)	53 (91.4)	43 (97.7)	0.210	6 (100.0)	3 (100.0)	3 (100.0)	8 (100.0)	4 (100.0)	4 (100.0)
Others	5 (4.9)	5 (8.6)	1 (2.3)		0 (0.0)	0 (0.0)	0 (0.0)	0 (0.0)	0 (0.0)	0 (0.0)
**Education (in years)**										
Illiterate	12 (12.8)	7 (13.5)	5 (11.9)	1	0 (0.0)	0 (0.0)	0 (0.0)	0 (0.0)	0 (0.0)	0 (0.0)
1–4	47 (50.0)	28 (53.8)	19 (45.2)	0.938	0 (0.0)	0 (0.0)	0 (0.0)	7 (87.5)	3 (75.0)	4 (100.0)
5–8	13 (13.8)	6 (11.5)	7 (16.7)	0.543	4 (66.7)	2 (66.7)	2 (66.7)	1 (12.5)	1 (25.0)	0 (0.0)
≥8	22 (23.4)	11 (21.2)	11 (26.2)	0.642	2 (33.3)	1 (33.3)	1 (33.3)	0 (0.0)	0 (0.0)	0 (0.0)
**Anatomical region of the bite**										
Upper limbs	7 (6.9)	3 (5.2)	4 (9.1)	0.438	0 (0.0)	0 (0.0)	0 (0.0)	0 (0.0)	0 (0.0)	0 (0.0)
Lower limbs	95 (93.1)	55 (94.8)	40 (90.9)		6 (100.0)	3 (100.0)	3 (100.0)	8 (100.0)	4 (100.0)	4 (100.0)
**Time from bite to medical assistance (in hours)**										
≤1	8 (11.4)	3 (6.8)	5 (19.2)	1	0 (0.0)	0 (0.0)	0 (0.0)	0 (0.0)	0 (0.0)	0 (0.0)
1–6	38 (54.3)	23 (52.3)	15 (57.7)	0.268	6 (100.0)	3 (100.0)	3 (100.0)	8 (100.0)	4 (100.0)	4 (100.0)
>6	24 (34.3)	18 (18.2)	6 (19.2)	0.078	0 (0.0)	0 (0.0)	0 (0.0)	0 (0.0)	0 (0.0)	0 (0.0)

Group A is the freeze-dried trivalent antivenom (FDTAV); Group B is available *Bothrops*, *Bothrops-Lachesis* and *Bothrops-Crotalus* AVs provided by the MoH (MoHA).

**Table 3:** Baseline laboratorial features of the included patients and comparison between experimental groups.

Variable	*Bothrops* bites (number, %)				*Lachesis* bites (number, %)			*Crotalus* bites (number, %)		
	Total	Group A	Group B	p	Total	Group A	Group B	Total	Group A	Group B
**Clotting time**										
Normal (0–10 min)	36 (35.3)	17 (29.3)	19 (43.2)	0.146	0 (0.0)	0 (0.0)	0 (0.0)	3 (37.5)	2 (50.0)	1 (25.0)
Abnormal (>10 min)	66 (64.7)	41 (70.7)	25 (56.8)		6 (100.0)	3 (100.0)	3 (100.0)	5 (62.5)	2 (50.0)	3 (75.0)
**Urea**										
Normal (15–40 mg/dL)	56 (54.9)	29 (50.0)	27 (61.4)	0.253	6 (100.0)	3 (100.0)	3 (100.0)	5 (62.5)	2 (50.0)	3 (75.0)
High (>40 mg/dL)	46 (45.1)	29 (50.0)	17 (38.6)		0 (0.0)	0 (0.0)	0 (0.0)	3 (37.5)	2 (50.0)	1 (25.0)
**Creatinine**										
Normal (0.5–1.2 mg/dL)	95 (93.1)	54 (91.4)	41 (95.5)	0.682	6 (100.0)	3 (100.0)	3 (100.0)	8 (100.0)	4 (100.0)	4 (100.0)
High (>1.2 mg/dL)	7 (6.9)	5 (8.6)	2 (4.5)		0 (0.0)	0 (0.0)	0 (0.0)	0 (0.0)	0 (0.0)	0 (0.0)
**K**^**+**^										
Normal (3.6–5.2 mmol/L)	83 (81.4)	48 (82.8)	35 (79.6)	0.700	…/*	…/*	…/*	…/*	…/*	…/*
Low (<3.6 mmol/L)	19 (18.6)	10 (17.2)	9 (20.4)		…/*	…/*	…/*	…/*	…/*	…/*
**Na**^**2+**^										
Normal (135–145 mmol/L)	81 (79.4)	45 (77.6)	36 (81.8)	0.609	…/*	…/*	…/*	…/*	…/*	…/*
High (>145 mmol/L)	21 (20.6)	13 (22.4)	8 (18.2)		…/*	…/*	…/*	…/*	…/*	…/*
**Lactate dehydrogenase**										
Normal (211–423 mg/dL)	3 (2.9)	2 (3.4)	1 (2.3)	0.728	…/*	…/*	…/*	…/*	…/*	…/*
High (>423 mg/dL)	99 (97.1)	56 (96.6)	43 (97.7)		…/*	…/*	…/*	…/*	…/*	…/*
**Leucocytes**										
Normal (4,000–10,000 mm^3^)	48 (47.0)	13 (22.4)	35 (79.5)	<0.001	…/*	…/*	…/*	…/*	…/*	…/*
High (>10,000 mm^3^)	54 (53.0)	45 (77.6)	9 (20.5)		…/*	…/*	…/*	…/*	…/*	…/*
**Hemoglobin**										
Normal (female: 12–16 g/dL; male: 13–18 g/dL)	43 (42.2)	26 (44.8)	17 (38.6)	0.511	…/*	…/*	…/*	…/*	…/*	…/*
Low (female: <12 g/dL; male: <13 g/dL)	59 (57.8)	32 (55.2)	27 (61.4)		…/*	…/*	…/*	…/*	…/*	…/*
**Aspartate transaminase**										
Normal (2–38 mg/dL)	53 (52.0)	25 (43.1)	27 (61.4)	0.073	6 (100.0)	3 (100.0)	3 (100.0)	6 (75.0)	3 (75.0)	3 (75.0)
High (>38 mg/dL)	49 (48.0)	33 (56.9)	17 (39.6)		0 (0.0)	0 (0.0)	0 (0.0)	2 (25.0)	1 (25.0)	1 (25.0)
**Alanine transaminase**										
Normal (2–44 mg/dL)	56 (54.9)	29 (50.0)	27 (61.4)	0.253	…/*	…/*	…/*	…/*	…/*	…/*
High (>44 mg/dL)	46 (45.1)	29 (50.0)	17 (38.6)		…/*	…/*	…/*	…/*	…/*	…/*
**Creatine phosphokinase**										
Normal (24–190 U/L)	49 (48.0)	28 (48.3)	21 (47.7)	0.956	6 (100.0)	3 (100.0)	3 (100.0)	5 (62.5)	2 (50.0)	3 (75.0)
High (>190 U/L)	53 (52.0)	30 (51.7)	23 (52.3)		0 (0.0)	0 (0.0)	0 (0.0)	3 (37.5)	2 (50.0)	1 (25.0)
**Fibrinogen**										
Normal (150–370 mg/dL)	25 (24.5)	14 (24.1)	11 (25.0)	0.920	0 (0.0)	0 (0.0)	0 (0.0)	4 (50.0)	3 (75.0)	1 (25.0)
Low (<150 mg/dL)	77 (75.5)	44 (75.9)	33 (75.0)		6 (100.0)	3 (100.0)	3 (100.0)	4 (50.0)	1 (25.0)	3 (75.0)
**Platelets**										
Normal (150.000–450.000/mm^3^)	85 (83.3)	49 (84.5)	36 (81.8)	0.721	…/*	…/*	…/*	…/*	…/*	…/*
Low (<150.000 mm^3^)	17 (16.7)	9 (15.5)	8 (18.2)		…/*	…/*	…/*	…/*	…/*	…/*
**International Normalized Ratio**										
Normal (1)	35 (34.3)	22 (37.9)	13 (29.5)	0.377	0 (0.0)	0 (0.0)	0 (0.0)	4 (50.0)	3 (75.0)	1 (25.0)
High (>1)	67 (65.7)	36 (62.1)	31 (70.5)		6 (100.0)	3 (100.0)	3 (100.0)	4 (50.0)	1 (25.0)	3 (75.0)
**Erythrocyte sedimentation rate**										
Normal (≤6 mm in the first hour)	2 (2.0)	0 (0.0)	2 (4.5)	0.109	…/*	…/*	…/*	…/*	…/*	…/*
Abnormal (>6 mm in the first hour)	100 (98.0)	58 (100.0)	42 (95.5)		…/*	…/*	…/*	…/*	…/*	…/*

Group A is the freeze-dried trivalent antivenom (FDTAV); Group B is available *Bothrops*, *Bothrops-Lachesis* and *Bothrops-Crotalus* AVs provided by the MoH (SLAV); …/*: Test not performed.

**Table 4:** Baseline clinical features of the included patients and comparison between experimental groups.

Variable	*Bothrops* bites (number, %)				*Lachesis* bites (number, %)			*Crotalus* bites (number, %)		
	Total	Group A	Group B	p	Total	Group A	Group B	Total	Group A	Group B
**Local manifestations**										
Local pain	98 (96.1)	56 (96.6)	42 (95.5)	0.777	6 (100.0)	3 (100.0)	3 (100.0)	8 (100.0)	4 (100.0)	4 (100.0)
Edema	98 (96.1)	54 (93.1)	44 (100.0)	0.076	6 (100.0)	3 (100.0)	3 (100.0)	8 (100.0)	4 (100.0)	4 (100.0)
Local bleeding	31 (30.4)	16 (27.6)	15 (34.1)	0.479	2 (33.3)	1 (33.3)	1 (33.3)	1 (12.5)	0 (0.0)	1 (25.0)
Erythema	38 (37.3)	25 (43.1)	13 (29.5)	0.161	0 (0.0)	0 (0.0)	0 (0.0)	0 (0.0)	0 (0.0)	0 (0.0)
Necrosis	2 (1.9)	2 (3.4)	0 (0.0)	0.213	0 (0.0)	0 (0.0)	0 (0.0)	0 (0.0)	0 (0.0)	0 (0.0)
Secondary infection	37 (36.3)	20 (34.5)	17 (38.6)	0.665	0 (0.0)	0 (0.0)	0 (0.0)	0 (0.0)	0 (0.0)	0 (0.0)
Blistering	3 (2.9)	2 (3.4)	1 (2.3)	0.728	0 (0.0)	0 (0.0)	0 (0.0)	0 (0.0)	0 (0.0)	0 (0.0)
Paresthesia	0 (0.0)	0 (0.0)	0 (0.0)	…	0 (0.0)	0 (0.0)	0 (0.0)	1 (12.5)	0 (0.0)	1 (25.0)
**Systemic manifestations**										
Bleeding	7 (6.9)	3 (5.2)	4 (9.1)	0.438	1 (16.7)	0 (0.0)	1 (33.3)	0 (0.0)	0 (0.0)	0 (0.0)
Vomiting	2 (2.0)	1 (1.7)	1 (2.3)	0.843	3 (50.0)	1 (33.3)	2 (66.7)	1 (12.5)	0 (0.0)	1 (25.0)
Myalgia	6 (5.9)	1 (1.7)	5 (11.4)	0.040	0 (0.0)	0 (0.0)	0 (0.0)	2 (25.0)	1 (25.0)	1 (25.0)
Nausea	12 (11.8)	6 (10.3)	6 (13.6)	0.609	0 (0.0)	0 (0.0)	0 (0.0)	0 (0.0)	0 (0.0)	0 (0.0)
Acute renal failure	7 (6.9)	5 (8.6)	2 (4.5)	0.420	0 (0.0)	0 (0.0)	0 (0.0)	0 (0.0)	0 (0.0)	0 (0.0)
Oliguria	4 (3.9)	3 (5.2)	1 (2.3)	0.455	0 (0.0)	0 (0.0)	0 (0.0)	1 (12.5)	0 (0.0)	1 (25.0)
Hematuria	4 (3.9)	2 (3.4)	2 (4.5)	0.777	0 (0.0)	0 (0.0)	0 (0.0)	0 (0.0)	0 (0.0)	0 (0.0)
Headache	0 (0.0)	0 (0.0)	0 (0.0)	…	3 (50.0)	1 (33.3)	2 (66.7)	1 (12.5)	1 (25.0)	0 (0.0)
Bradycardia	0 (0.0)	0 (0.0)	0 (0.0)	…	3 (50.0)	2 (66.7)	1 (33.3)	0 (0.0)	0 (0.0)	0 (0.0)
Hypotension	0 (0.0)	0 (0.0)	0 (0.0)	…	4 (66.7)	2 (66.7)	2 (66.7)	0 (0.0)	0 (0.0)	0 (0.0)
Abdominal pain	0 (0.0)	0 (0.0)	0 (0.0)	…	2 (33.3)	1 (33.3)	1 (33.3)	0 (0.0)	0 (0.0)	0 (0.0)
Blurred vision	0 (0.0)	0 (0.0)	0 (0.0)	…	2 (33.3)	0 (0.0)	2 (66.7)	5 (62.5)	1 (25.0)	4 (100.0)
Palpebral ptosis	0 (0.0)	0 (0.0)	0 (0.0)	…	0 (0.0)	0 (0.0)	0 (0.0)	4 (50.0)	2 (50.0)	2 (50.0)
Diplopia	0 (0.0)	0 (0.0)	0 (0.0)	…	0 (0.0)	0 (0.0)	0 (0.0)	6 (75.0)	3 (75.0)	3 (75.0)
Ophtalmoplegia	0 (0.0)	0 (0.0)	0 (0.0)	…	0 (0.0)	0 (0.0)	0 (0.0)	1 (12.5)	0 (0.0)	1 (25.0)
Dizziness	0 (0.0)	0 (0.0)	0 (0.0)	…	0 (0.0)	0 (0.0)	0 (0.0)	1 (12.5)	0 (0.0)	1 (25.0)

Group A is the freeze-dried trivalent antivenom (FDTAV); Group B is available *Bothrops*, *Bothrops-Lachesis* and *Bothrops-Crotalus* AVs provided by the MoH (SLAV).

In general, epidemiological, clinical and laboratorial characteristics were similarly distributed in the two study groups within *Bothrops*, *Lachesis* and *Crotalus* bitten groups.

### Efficacy endpoints analysis

**Fig 2:**
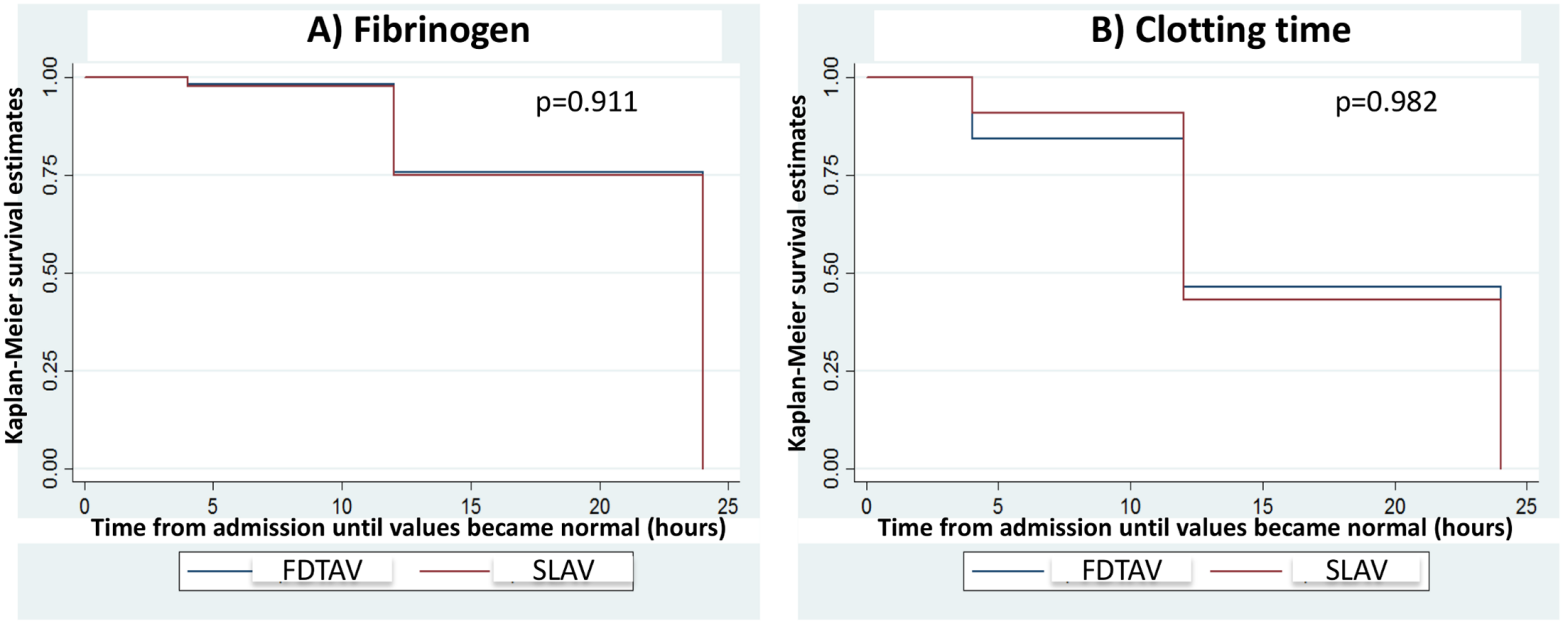
Survival analysis of the primary efficacy endpoint. Time until fibrinogen (Part A) and clotting time (Part B) reaching normal values over follow-up of patients bitten by *Bothrops* snakes presented no significant difference between freeze-dried trivalent antivenom (FDTAV) and Ministry of Health standard liquid antivenoms (SLAVs) treated groups.

**Fig 3:**
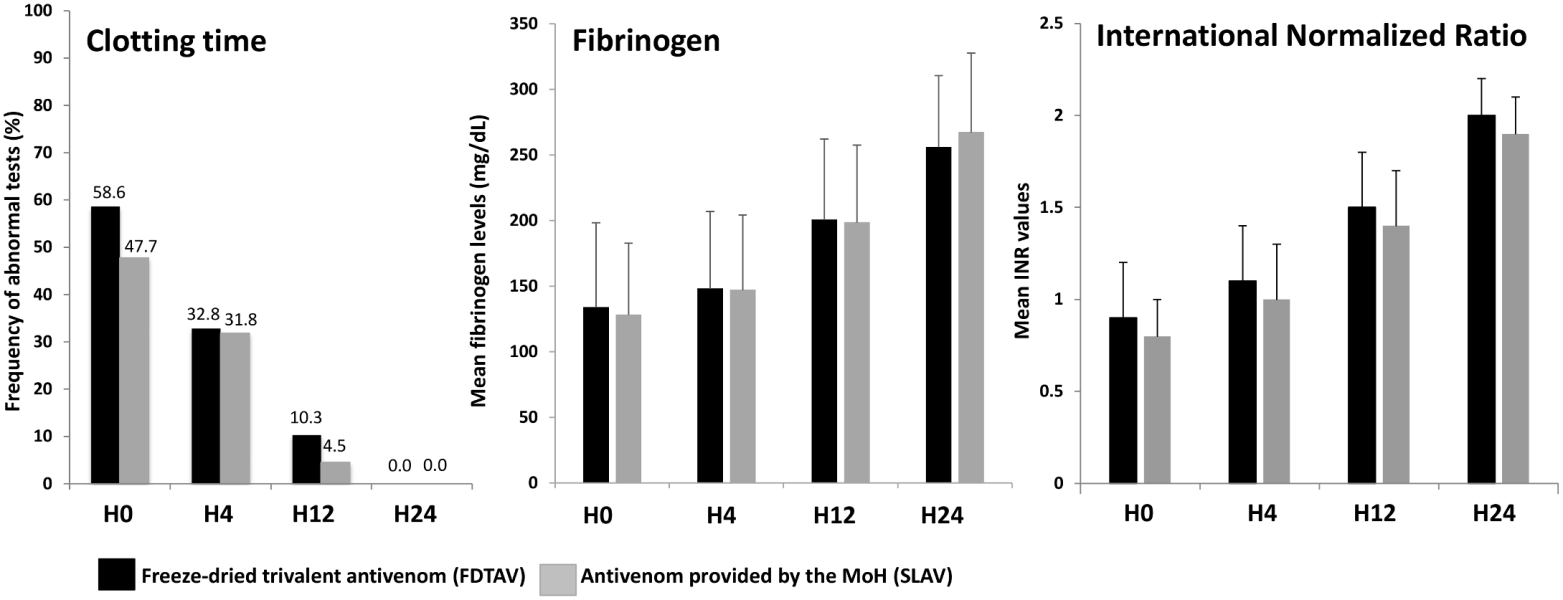
Primary efficacy endpoint analysis in *Bothrops* snakebites. Clotting time, fibrinogen and INR levels 24 hours after AV therapy in freeze-dried trivalent antivenom (FDTAV) and Ministry of Health standard liquid antivenoms (SLAVs) treated groups.

There were no differences between FDTAV and MoH AV treated groups in the time until patient admission for fibrinogen (p = 0.911) and clotting time (p = 0.982), both reaching normal values over follow-up in patients bitten by *Bothrops* snakes (Fig 2). Clotting time, fibrinogen and INR presented normal values 24 hours after AV therapy, for both antivenoms (Fig 3). Moreover, creatinine, liver transaminases, creatine phosphokinase and the other laboratorial markers were normal 24 hours after admission in FDTAV and SLAV treated groups.

For *Lachesis* snakebites, fibrinogen, clotting time and INR presented normal values 24 hours after AV therapy for both antivenoms (S3 File).

Regarding *Crotalus* snakebites, fibrinogen, clotting time, INR, and creatine phosphokinase presented normal values 24 hours after AV therapy in FDTAV and SLAV treated groups (S4 File). Signs and symptoms such as blurred vision, palpebral ptosis, diplopia and ophtalmoplegia disappeared within the first 24 hours of follow-up. The other laboratorial parameters also returned to normal ranges within the first 24 hours of follow-up for both groups. Renal failure was not seen in this group of patients.

No patient required a second AV dose after the initial dose because of persistent coagulopathy and no local or coagulopathy recurrence was recorded among the patients. All clinical, both locally and systemically, and laboratorial evaluations remained normal at D7 and D15 in both treatment groups.

### Safety endpoint analysis

A total of 23 patients presented early adverse events after AV therapy (19.8%). The most common were urticaria (13.8%), pruritus (11.2%), facial flushing (3.4%) and vomiting (3.4%). There was no significant difference in the frequency of early adverse events between patients treated with FDTAV and SLAV (Table 5). All adverse events were mild and all signs and symptoms of early adverse reactions ceased within 48 hours from management. Late adverse events were not observed at D7 and D15 clinical evaluations. Patients were advised to report these after D15, however no patient returned to the hospital or contacted the study physician by phone complaining about possible late adverse events after that day.

**Table 5:** Adverse events reported in the safety study and comparison between the experimental groups.

Adverse event	Total (n = 116)		Group A (n = 65)		Group B (n = 51)		p
	Number	%	Number	%	Number	%	
Any adverse event	23	19.8	11	16.9	12	23.5	0.388
Urticaria	16	13.8	11	16.9	5	9.8	0.286
Pruritus	13	11.2	8	12.3	5	9.8	0.691
Facial flushing	4	3.4	3	4.6	1	2.0	0.811
Vomiting	4	3.4	2	3.1	2	3.9	>0.999
Headache	2	1.7	0	0.0	2	3.9	0.821
Dyspnea	2	1.7	1	1.5	1	2.0	>0.999
Chills	2	1.7	1	1.5	1	2.0	>0.999
Nasal congestion	2	1.7	1	1.5	1	2.0	>0.999
Nausea	1	0.9	1	1.5	0	0.0	0.632
Conjunctival hyperemia	1	0.9	1	1.5	0	0.0	0.632
Pharyngeal irritation	1	0.9	1	1.5	0	0.0	0.632

Group A is the freeze-dried trivalent antivenom (FDTAV); Group B is available *Bothrops*, *Bothrops-Lachesis* and *Bothrops-Crotalus* AVs provided by the MoH (SLAV).

### Quality control and stability results

**Fig 4:**
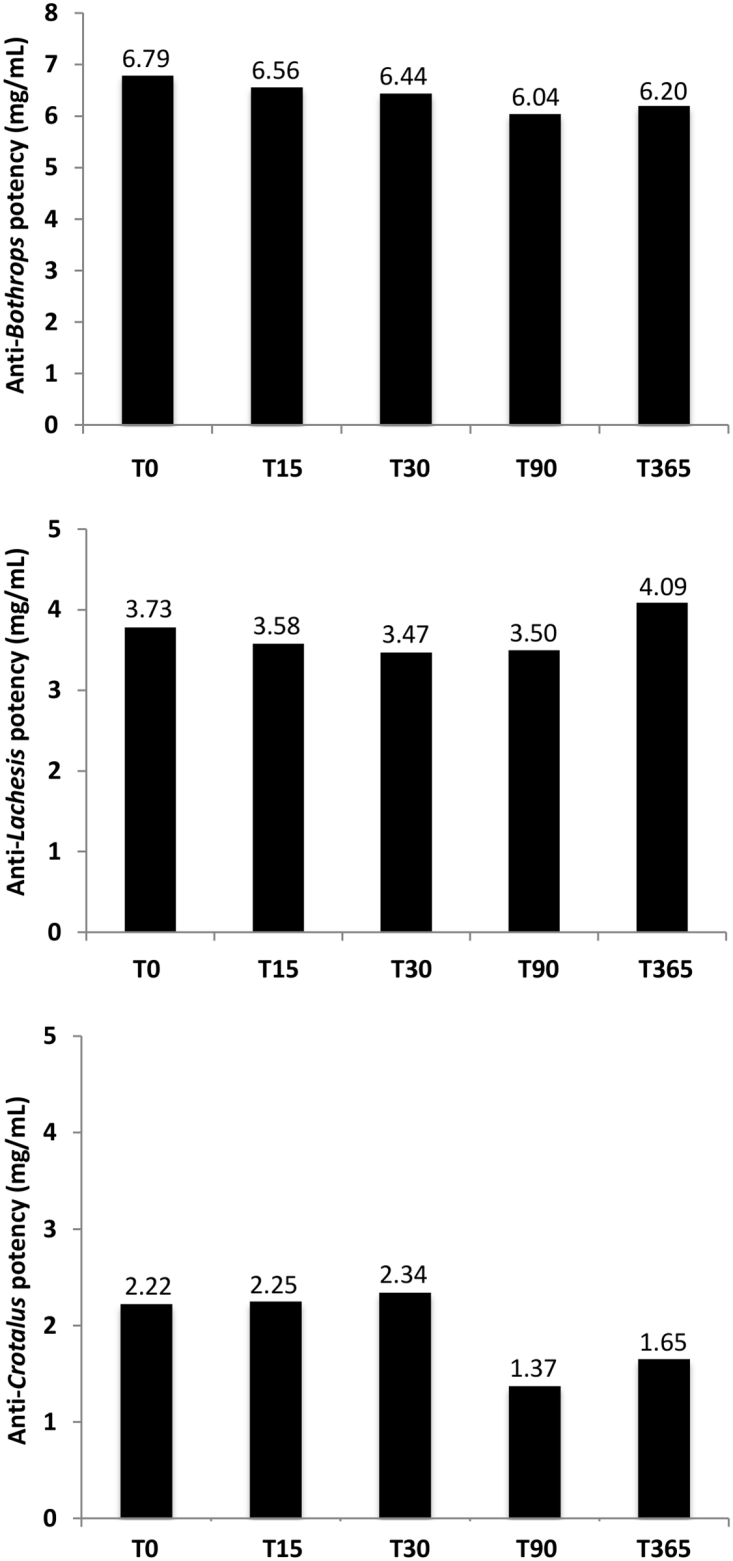
Thermostability evaluations of the freeze-dried trivalent antivenom (FDTAV) at 56°C over one year. Antivenom potency for neutralizing *Bothrops*, *Lachesis* and *Crotalus* activities showed no significant decrease compared to baseline over one year of thermostability evaluations at 56°C.

FDTAV physicochemical analysis showed a total protein concentration of 4.63g%, with 80% of F(ab’)_2_ immunoglobulins. FDTAV was sterile, atoxic and apyrogenic. Phenol concentration was <3,500 ppm. Antivenom potency for neutralizing *Bothrops*, *Lachesis* and *Crotalus* activities showed no significant decrease compared to baseline potency over one year of thermostability evaluations at 56°C (Fig 4). No visual alterations in turbidity and no aggregates were observed over time and before AV administration.

## Discussion

Antivenoms are the only specific treatment for treating most of the snakebite envenomings effects, and play a crucial role in minimizing mortality and morbidity. However, to be part of the primary health care package where snakebites occur, there is an urgent need to ensure availability of safe, effective and affordable AVs, namely in developing countries [16]. Moreover, innovation in the logistics of AV distribution is also a priority. In Brazil, for instance, current AVs require conservation in adequate facilities (2° to 8°C), which are not always available in remote settings [4]. Although freeze-dried formulations may become a good alternative for the production of more stable AVs in regions of the world where high temperatures are common and the cold chain is poor, such in the Brazilian Amazon, there is a very limited body of published literature on stability, clinical efficacy and safety for freeze-dried AVs. A main concern is the conservation of the freeze-dried AV pharmacokinetic properties and further neutralizing potency of the antibodies submitted to lyophilization [25]. Although positive results from thermal characterization of the effect of freeze-drying on equine antibodies and the effectiveness of stabilizers in freeze-dried AVs have been reported [18,19], to the best of our knowledge this is the first clinical trial aiming to compare the safety and efficacy of a freeze-dried AV with a liquid formulation. This study did not include data on the possible increase in the cost of AV production due to the freeze-drying process. Future cost studies to address this will be essential.

### Efficacy analysis

Our results showed that in both groups neutralization of venom-induced bleeding and coagulopathy invariably ceased, and blood coagulability was restored over 24 hours of follow-up of all patients. Since hemostasis disorders have been related to severity and deaths in viperid envenomations, restoration of blood coagulability has been proposed as a relevant target endpoint in snakebite clinical trials [26,27]. Actually, systemic manifestations of viperid snakebites are characterized by bleeding, coagulopathy associated with defibrinogenation and incoagulability, with the potential of evolving to hypovolemia and further potentially life-threatening conditions such as hypotension, cardiovascular shock and acute kidney injury in severe cases [5,28–30]. Our results indicate that an improvement in coagulation status might be expected within 6–12 hours in most patients and within 24 hours in nearly all patients receiving a single dose of AV [6,31–33]. Blood coagulability should be monitored after the initial AV dose and further doses of AV should be administered, if necessary, until coagulability is restored [31,33,34]. Abnormal renal function is also fairly common in viperid envenomings [5,35,36]. Both study groups presented serum creatinine concentrations that restored to normal values within the first 24 hours of follow-up of *Bothrops* snakebites. However, some patients admitted with renal failure may evolve without improvement of kidney injury even after AV administration, as injury was triggered before AV administration [35,36]. This needs to be taken in consideration in the interpretation of renal failure as an endpoint in *Bothrops* bites.

*Crotalus* venom may cause systemic myotoxicity, increasing serum levels of creatine kinase [12]. In addition, the levels of muscle enzymes increase even after properAV therapy [12,37,38]. Likewise, even patients who receive proper amounts of antivenom can progress with renal damage because the mechanisms of injury are triggered after snakebite and before the antivenom administration. In this study, however, no patient presented renal failure at admission and normal creatine kinase levels were seen at 24 hours of follow-up of *Crotalus* snakebites. All patients recovered from coagulopathy within 24 hours after therapy with FDTAV or SLAV. Although some neurological manifestations observed in *Crotalus* envenomation do not usually improve rapidly after AV administration [12], in this trial signs and symptoms such as blurred vision, palpebral ptosis, diplopia and ophtalmoplegia were suppressed within the first 24 hours of follow-up.

In this study, a second dose of AV was not necessary, since no patient presented the blood totally incoagulable 12 hours after the initial dose or coagulopathy recurrences. Indeed, persistent or recurrent venom antigenemia and even recurrent coagulopathy may be present within 12–48 hours in severe envenoming [39,40], but severe cases were not included in this trial. Moreover, differences in the efficacy of the available antivenom also explain dissimilar rates of resolution of venom-induced coagulopathy, as observed using AVs from different Latin American countries [41–44]. Furthermore, venom composition is known to vary significantly among snakes of different species further reflecting in AV efficacy [14,45]. Data concerning *Lachesis and Crotalus* species have limitations in terms of efficacy because of the small number due to the rarity of these cases in the Amazon.

### Safety analysis

Although the literature suggests that the amount of heterologous proteins is related to the frequency and intensity of adverse reactions [46], we did not observe a significant increase in the incidence of adverse events in FDTAV compared to SLAV. Although both AVs were manufactured from the same pool of hyperimmune plasma, one concern of this study is the possible formation of insoluble aggregates during the freeze-drying process with further incomplete reconstitution, which has been associated with the development of adverse events during administration [47]. One hypothesis for the negligible insoluble aggregates formation in the FDTAV is the use of sucrose in the stabilization of the lyophilized AV. The protective effect of polyols like sorbitol and mannitol in the development of turbidity in liquid AVs has already been reported [48–50]. More recently, the use of sucrose in the stabilization of liquid or freeze-dried AVs has been described as a better stabilizer than mannitol and sorbitol in the FDTAV formulation [18,19].

Early AV reactions were mild and were promptly reversed by treatment with adrenaline and antihistamines. The incidence of early adverse reactions, all mild and not life-threatening, was similar to two other trials from Brazil and Colombia [6,39]. However, generally this frequency was lower than the results from other trials carried out in South America with *Bothrops* bitten patients, which reported incidences ranging from 25 to 87% [31–33,40,43,51]. Although antihistamines appear to be of no obvious benefit in preventing acute reactions from antivenoms [52–54], the pre-sorotherapy regimen used in this study (IV hydrocortisone, IV cimetidine and oral dexchlorpheniramine) may have also contributed to the low incidence of early adverse reactions. Late adverse events were not observed in this trial, in agreement with previous studies showing their absence [8,31–33,40,43,51] or low incidence [55]. Although no patient returned to the hospital or contacted the study physician complaining about possible late adverse events after D15, patients treated with snake AVsmay present adverse reactions after this day. On-site follow-up completion on this day may represent a limitation of this study that may underestimate the frequency of reactions. A concern for the use of AVs formulated with sucrose has been the possibility of complicating or producing some renal damage [56], but renal function was normal for all patients after 24 hours of follow-up, indicating that the dosage employed was safe.

### Reconstitution test

Previous studies reported prolonged reconstitution times of 30 and 90 minutes for some lyophilized AVs [57,58], delaying the time of AV administration, wich is a factor related to poor prognosis of snakebites envenomation [5]. In this study, however, a complete dissolution was observed visually as the FDTAV vials were gently agitated by hand for one minute, in agreement with previous studies where other freeze-dried proteins showed short reconstitution times (lower than 5 minutes) [18,59,60], suggesting maintenance of the stability and neutralizing activity [60,61].

### Stability results

Successful freeze-drying of AVs already used routinely in some continents (despite the lack of published clinical trials) requires the conservation of the physicochemical stability and neutralizing potency of the antibodies over time at room temperature [18]. In this study, AV potency for neutralizing *Bothrops*, *Lachesis* and *Crotalus* venom activities showed no significant decrease comparing to baseline over one year of thermostability evaluations at 56°C. Osmolytes such as sucrose or sorbitol are capable of stabilizing antibodies at high temperatures with no significant perturbation in their secondary structure or in their affinity to snake venoms [48,49]. Liquid AVs with sorbitol prevent the appearance of turbidity after one year storage at 37°C, but show a partial loss in neutralizing potency in these conditions [50]. When tested, anti-α-cobratoxin single domain antibodies added with disulfide bonds at 1 mg/mL at a range of elevated temperatures for an hour retainednearly 100% of their initial binding activity [62].

### Final remarks

In this study, FDTAV and SLAVs presented the same efficacy based on clinically relevant outcomes in *Bothrops*, *Lachesis* and *Crotalus* bites. Furthermore, there was no significant difference in the incidence of early adverse reactions between patients treated with FDTAV and SLAVs. Only mild early adverse reactions were reported. This freeze-dryed formulation may become a good alternative for the production of more stable AVs in regions of the Amazon where high temperatures are common and the cold chain is poor. Our results indicate such a product should be attainable for a phase III trial.

## Supporting information

S1 Checklist CONSORT checklist. (DOC)

S1 Dataset Study database. (XLSX)

S1 Appendix Ethics approval document. (DOCX)

S2 Appendix Original study protocol. (PDF)

S1 File Methods employed for estimating AV potency. (DOCX)

S2 File Baseline laboratorial features of the included patients and comparison of means between experimental groups in *Bothrops* snakebites. (DOCX)

S3 File Primary efficacy endpoint analysis in *Lachesis* snakebites. For *Lachesis* snakebites, fibrinogen, clotting time and INR presented normal values 24 hours after AV therapy, for freeze-dried trivalent antivenom (FDTAV) and Ministry of Health standard liquid antivenoms (SLAV) treated groups. Creatinine levels were normal since the admission.
(PPTX)

S4 File Primary efficacy endpoint analysis in *Crotalus* snakebites. For *Crotalus* snakebites, fibrinogen, clotting time, INR, and creatine phosphokinase presented normal values 24 hours after AV therapy in FDTAV and Ministry of Health standard liquid antivenoms (SLAV) treated groups.
(PPTX)
