# Correction: Safety and efficacy of a freeze-dried trivalent antivenom for snakebites in the Brazilian Amazon: An open randomized controlled phase IIb clinical trial

**DOI:** 10.1371/journal.pntd.0010031

**Published:** 2021-12-10

**Authors:** Iran Mendonça-da-Silva, Antônio Magela Tavares, Jacqueline Sachett, José Felipe Sardinha, Lilian Zaparolli, Maria Fátima Gomes Santos, Marcus Lacerda, Wuelton Marcelo Monteiro

The following information is missing from the Funding statement: This work was supported by CAPES/Fapeam.
