# Supplementary material for: Safety and efficacy of a freeze-dried trivalent antivenom for snakebites in the Brazilian Amazon: An open randomized controlled phase IIb clinical trial
Source: PLoS Negl Trop Dis. 2017 Nov 27;11(11):e0006068. doi: 10.1371/journal.pntd.0006068 (PMC5720814; doi:10.1371/journal.pntd.0006068)
Supplement: S2 Appendix — (PDF) [file pntd.0006068.s004.pdf]

PROJETO DE PESQUISA

AVALIAÇÃO CLÍNICO-TERAPÊUTICA DO SORO ANTIOFÍDICO TRIVALENTE LIOFILIZADO (BOTRÓPICO-LAQUÉTICO-CROTÁLICO)

INSTITUIÇÃO RESPONSÁVEL PELO PROJETO

INSTITUTO DE BIOLOGIA DO EXÉRCITO (RIO DE JANEIRO)

PARCERIA PARA EXECUÇÃO

FUNDAÇÃO DE MEDICINA TROPICAL DO AMAZONAS

HOSPITAL GERAL DE RORAIMA

COORDENADOR GERAL DE PESQUISA

IRAN MENDONÇA DA SILVA

(PESQUISADOR DO INSTITUTO DE BIOLOGIA DO EXÉRCITO)

MINISTÉRIO DA DEFESA
EXÉRCITO BRASILEIRO
CML - 1ª RM
INSTITUTO DE BIOLOGIA DO EXÉRCITO
Comitê de Ética em Pesquisa

FOLHA DE ROSTO PARA PESQUISA ENVOLVENDO SERES HUMANOS

Projeto de Pesquisa: **AVALIAÇÃO CLÍNICO-TERAPÊUTICA DO SORO ANTIOFÍDICO TRIVALENTE (BOTRÓPICO-LAQUÉTICO-CROTÁLICO) LIOFILIZADO**

2. Área do Conhecimento (Conforme relação no verso): MEDICINA	3. Código: 4.01	4. Nível (Para áreas do conhecimento 2 a 4): "D", "T" e "E"
5.Área(s) Temática(s) (Conforme relação no verso): Medicamentos	6. Código(s): 3	7. Fase (Para área temática 3): 2

PESQUISADOR RESPONSÁVEL

9. Nome: IRAN MENDONÇA DA SILVA				
10. Identidade: 011117544-4 EB	11. CPF.: 929290528-72	17.Endereço (Rua, nº): Rua Francisca Matos, 560 – Bancários – Ilha do Governador		
12. Nacionalidade: Brasileiro	13. Profissão: Médico	18. CEP: 21910-055	19. Cidade: Rio de Janeiro	20.U.F.: RJ
14. Maior Titulação: Pós-graduação (Mestrado)		21. Fone: 021-XX-24673850	22. Fax: 021-XX-38603510	
16. Instituição a que pertence: Instituto de Biologia do Exército			23. E.mail: med1od2@aol.com	

Termo de Compromisso:
Declaro que conheço e cumprirei os requisitos da Res. CNS 196/96 e suas Complementares e aceito as responsabilidades pela condução científica do projeto acima.

Data: 28 / 04 / 2004

Assinatura

INSTITUIÇÃO ONDE SERÁ REALIZADO

24. Nome: Instituto de Biologia do Exército	28. Endereço (Rua, nº): Rua Francisco Manuel, 102 - Benfica		
25. Unidade/Órgão: Centro de Pesquisa Gen. Dr. Ismael da Rocha	29. CEP: 20911-270	30. Cidade: Rio de Janeiro	31. U.F.: RJ
26. Projeto Multicêntrico: Sim ( ) Não (x) — Participação Estrangeira: ( ) Sim (x) Não	32. Fone: 021-XX-38600442 Ramal 228	33. Fax.: 021-XX-38603510	

27. Outras Instituições participantes, inclusive, estrangeiras (Use folha anexa SN): Fundação de Medicina Tropical do Amazonas e Instituto Butantan (São Paulo)

Termo de Compromisso:
Declaro que conheço e cumprirei os requisitos da Res. CNS 196/96 e suas Complementares e como esta instituição tem condições para o desenvolvimento deste projeto, autorizo sua execução

Nome: Benedito Domício Ferrari
Cargo: Diretor do IBEx
Data: 19 / JUL / 04

Assinatura

PATROCINADOR — **Não se aplica (x)**

34. Nome:	37. Endereço		
35. Responsável:			
36. Cargo/Função:	38. CEP:	39. Cidade:	40. UF
	41. Fone:	42. Fax:	

COMITÊ DE ÉTICA EM PESQUISA - CEP

43. Data de Entrada: 13 / 08 / 2003	44. Protocolo: 001 - 03	45.Conclusão: Aprovado (x) Data: 18 / 09 / 2003	46. Não Aprovado ( ) Data: ____/____/____

47. Relatório(s) do Pesquisador responsável previsto(s) para:
30 / 06 / 2005   30 / 12 / 2005

Encaminho a CONEP:
48. Os dados acima para registro (x)
49. O projeto para apreciação ( )
50. Data: 06 / 12 / 2004

51. Coordenador/Nome
Assinatura

Anexar o parecer consubstanciado

COMISSÃO NACIONAL DE ÉTICA EM PESQUISA - CONEP

52. Protocolo	54. Registro no banco de dados:
53.Data Recebimento:	55. Observações:

AVALIAÇÃO CLÍNICO-TERAPÊUTICA DO SORO ANTIOFÍDICO TRIVALENTE
(BOTRÓPICO- LAQUÉTICO- CROTÁLICO) LIOFILIZADO

PROJETO DE PESQUISA

Coordenação do Projeto: Instituto de Biologia do Exército (IBEx – Rio de Janeiro)

Pesquisadores do IBEx

Maj Med Iran Mendonça da Silva (Chefe da Seção de Vigilância Epidemiológica)

Cap Farm Maria de Fátima dos Santos Gomes (Adjunto ao Centro de Pesquisas)

2º Ten Lílian de Amorim Zaparolli (Seção de Vigilância Epidemiológica)

Pesquisadores do Instituto Butantan

Drª Hisako Gondo Higashi (Diretora da Divisão de Desenvolvimento Tecnológico e Produção)

Dr. Rosalvo Guidolim (Liderança Científica)

Dr. José Roberto Marcelino (Chefe da Seção de Produção de Plasma Hiperimune)

Dr. José Amaral Prado (Chefe da Seção de Liofilização)

Drª Josefina Farina de Moraes (Bióloga)

Grupo Colaborativo na Execução do Projeto

Ten Cel Med Renildo Sérgio Batista dos Anjos (Hospital de Guarnição de Tabatinga – AM)

Maj Med Joner Moisés Cunha Patrocínio (Hospital de Guarnição de São Gabriel da Cachoeira – AM)

Maj Med José Ailton Martins de Souza (Posto Médico de Guarnição de Boa Vista - RR)

Drª. Kátia Cristina Barbaro (Laboratório de Imunopatologia – Instituto Butantan – SP)

Dr. Antônio de Matos Magela (Gerência do Pronto Atendimento da Fundação de Medicina Tropical do Amazonas – AM)

Dr. José Felipe Jardim Sardinha (Gerência de Diagnóstico da Fundação de Medicina Tropical do Amazonas – AM)

Drª. Camila Billia Flora (Médica do Hospital Geral de Roraima – RR)

Dr. Jean Cláudio Colares Sales (Médico da Unidade Hospitalar Mista de Borba – AM)

Dr. Jean Francisco Ventura Samonek (Gerência de Animais Peçonhentos da Fundação de Medicina Tropical do Amazonas – AM)

Rio de Janeiro, 15 de Setembro de 2003

ÍNDICE

INTRODUÇÃO....................3
HISTÓRICO....................4
RELEVÂNCIA DO ESTUDO....................5
DESENVOLVIMENTO TECNOLÓGICO....................5
LIOFILIZAÇÃO....................5
OBJETIVO GERAL....................7
OBJETIVOS ESPECÍFICOS....................7
MÉTODOS....................7
CRITÉRIOS DE ELEGIBILIDADE....................8
CRITÉRIOS DE EXCLUSÃO....................8
SOROTERAPIA....................8
SORO UTILIZADO NO ESTUDO....................8
PLANO AMOSTRAL....................9
RECRUTAMENTO DE PARTICIPANTES....................9
COLETA DE DADOS....................10
TESTES LABORATORIAIS....................10
PLANO DE ANÁLISE DOS DADOS....................11
CONSIDERAÇÕES ÉTICAS....................11
CRONOGRAMA DA PESQUISA....................14
ORÇAMENTO DA PESQUISA....................14
MATERIAL....................15
REFERÊNCIAS BIBLIOGRÁFICAS....................17

INTRODUÇÃO

Os acidentes por ofídios nos países em desenvolvimento têm grande importância em Saúde Pública em virtude de sua incidência e gravidade e, possíveis seqüelas que podem causar. No Brasil, o Ministério da Saúde tornou obrigatória, a partir de 1986, a notificação dos acidentes ofídicos, tendo sido notificados no ano de 2000, de junho a dezembro, 8.574 casos. No período de Janeiro de 1987 a dezembro de 1993 foram notificados 143.836 casos, o que representa uma média de 20.000 casos/ano para o país. Os dados foram obtidos a partir do Sistema de Vigilância Epidemiológica, implantado nas unidades federadas do país, apesar de que constatamos ainda uma evidente subnotificação, na maioria dos estados brasileiros. A maioria das notificações procede das Regiões Sudeste e Sul, as mais populosas do país e que contam com melhor organização de serviços de saúde e sistema de informação.

A distribuição sazonal dos casos para as Regiões Sul, Sudeste e Centro-Oeste, mostra um incremento dos acidentes no período de setembro a março. Na Região Nordeste, aumentam de janeiro a maio, enquanto que na Região Norte, principalmente no Estado do Amazonas, a ocorrência dos acidentes ofídicos mostra-se maior no período das chuvas (novembro a maio), particularmente na época de cheia dos rios em fevereiro (IMT- AM,1995).

A ocorrência do acidente ofídico está geralmente relacionada a fatores climáticos e ao aumento das atividades no setor agropecuário (preparo da terra, plantio e colheita). Essas observações parecem reforçar a idéia de que o acidente ofídico é um acidente de trabalho, uma vez que sua incidência coincide com o deslocamento do trabalhador rural para as suas atividades. Além disso, esses indivíduos não utilizam, de maneira adequada, a indumentária protetora, como sapatos, botas, perneiras e luvas. Em cerca de 53% das notificações, a faixa etária acometida situou-se entre 15-49 anos, o que corresponde ao grupo de idade onde mais concentra a força de trabalho. Aproximadamente 70% dos pacientes acometidos são do sexo masculino, o que é justificado pelo fato do homem desempenhar com mais freqüência atividades de trabalho fora da moradia, onde os acidentes ofídicos habitualmente ocorrem.

Há quatro gêneros de ofídios com importância médica no Brasil: *Bothrops* (incluindo *Bothriops* e *Porthidium*), *Crotalus*, *Lachesis* (pertencente à família Viperidae) e *Micrurus* (família Elapidae). O acidente botrópico é o de maior importância epidemiológica no país, pois é responsável por cerca de 90% dos acidentes por ofídios peçonhentos. Existem cerca de 30 espécies, distribuídas por todo o território nacional, tendo como habitat preferencial, ambientes úmidos das matas e áreas cultivadas. O acidente crotálico é responsável por 8% dos acidentes no Brasil. Há apenas uma espécie e é encontrada em todo o país, à exceção de áreas florestais e zona litorânea. São encontradas em campos, áreas secas, arenosas e pedregosas. Na Amazônia são encontradas em algumas regiões onde existem áreas de campos como na Ilha de Marajó, Santarém, norte do Pará, Humaitá, norte do Amapá e Roraima. Apresenta o maior coeficiente de letalidade devido à freqüência com que evolui para insuficiência renal aguda. O acidente

laquético representa em torno de 1,5% dos acidentes no Brasil. Há uma única espécie e habitam principalmente áreas florestais da Amazônia, Mata Atlântica e alguns enclaves de matas úmidas do Nordeste. O acidente elapídico representa em torno de 0,5% dos acidentes no Brasil. O gênero *Micrurus* compreende 18 espécies distribuídas por todo território nacional. Pode evoluir com insuficiência respiratória aguda, causa de óbito neste tipo de envenenamento.

A letalidade no Brasil está próxima de 0,5%, sendo que a causa da maioria dos óbitos ocorre no grupo de pacientes atendidos 6 ou mais horas após o acidente. Portanto, o tempo entre o acidente e a administração do soro, é sem dúvida alguma, o fator mais importante para o prognóstico do caso.

HISTÓRICO

As pesquisas de Sewal, de Kaufmann, de Physalix, de Bertrand e principalmente as de Calmette estabeleceram em bases científicas a noção de imunidade, em relação ao veneno ofídico. Mais do que isto, estes últimos pesquisadores, trabalhando em 1894, quando já eram conhecidos os trabalhos de Behring e Kitasato que firmaram as bases da soroterapia em relação à difteria e ao tétano, provaram que o soro dos animais vacinados contra a peçonha possuía também uma substância antitóxica capaz de neutralizar os efeitos do veneno e de transmitir imunidade passiva aos animais não preparados. Calmette conseguiu hiperimunizar grandes animais para o preparo do soro antiofídico, servindo-se principalmente do veneno de cobra indiana (*Naja tripudians*). Vital Brazil foi o primeiro a demonstrar a especificidade dos antivenenos. A partir desta descoberta, Vital Brazil iniciou em 1901, no Instituto Butantan, o preparo de antivenenos mono e polivalentes, os soros anticrotálico e antibotrópico para uso no Brasil. Esta sua orientação de preparação dos soros mono e polivalentes, para uso em uma determinada região, é adotada no mundo inteiro nos dias atuais.

Embora a metodologia do preparo dos soros antiofídicos não tenha avançado muito desde a sua descoberta, procedimentos de purificação e modos de utilização foram modificados consideravelmente. Os soros heterólogos são concentrações de imunoglobulinas obtidas através de soros de cavalos imunizados com o veneno. Os antivenenos são utilizados com o objetivo de neutralizar as atividades sistêmicas e locais dos venenos. A soroterapia antiveneno é a única medida específica para o tratamento adequado dos pacientes picados pela maioria dos animais peçonhentos.

RELEVÂNCIA DO ESTUDO

No Brasil, os laboratórios que produzem esses imunoderivados são o Instituto Butantan (São Paulo), Fundação Ezequiel Dias (Minas Gerais), Instituto Vital Brazil (Rio de Janeiro) entre outros. Os soros são produzidos em formulação líquida, envasadas em ampolas, com apresentação mono ou bivalentes, devendo ser armazenados à temperatura de 2 a 8 graus Celsius positivos, sendo sua validade em geral, de 2 a 3 anos. Entretanto para os países tropicais, a Organização Mundial de Saúde recomenda que os soros sejam apresentados na forma liofilizada.

Baseado neste contexto, o Instituto Butantan em parceria com o Instituto de Biologia do Exército iniciou a produção no ano de 2000, dos primeiros lotes de soro antiofídico liofilizado trivalente (botrópico-laquético-crotálico), apresentados em caixas com 5 frascos contendo cada um, duas doses, suficientes para neutralizar no mínimo, 100 mg, 60 mg, e 30 mg dos venenos de referência de *Bothrops jararaca*, *Lachesis muta* e *Crotalus durissus terrificus*, respectivamente (soroneutralização em camundongos). Acompanhando o produto, 5 ampolas de 20 ml de diluente para soro. De acordo com experimentos realizados pelo Instituto Butantan o produto liofilizado possui excelente termoestabilidade em ambientes de elevadas temperaturas - 56 graus Celsius, por um período de 12 meses – (Anexo I), sendo assim dispensada a necessidade de cuidados com refrigeração, tal como ocorre com o soro líquido convencional. Desta forma torna-se extremamente útil e prático para aplicação em pontos estratégicos do território nacional, principalmente na Região Amazônica, atendendo as tropas militares operacionais que realizam manobras, exercícios no terreno e acampamentos, bem como populações indígenas, ribeirinhas e trabalhadores rurais, distantes dos centros de atendimento médico.

Após a liofilização do produto foram realizados testes de controle interno de qualidade (esterilidade, pirogênico, inocuidade, potência e de características físico-químicas) e externo no INCQS/Fiocruz, ambos com resultados satisfatórios (Anexo II). Em uma segunda etapa serão realizados testes em humanos, propósito este do nosso estudo, atendendo as normas da Resolução nº196, de 10 de outubro de 1996 e da Resolução nº 251, de 07 de agosto de 1997, ambas do Conselho Nacional de Saúde, por se tratar de nova associação de substâncias (trivalente) e forma farmacêutica (liofilizada), diferentes daquelas estabelecidas quando da autorização do registro.

DESENVOLVIMENTO TECNOLÓGICO

LIOFILIZAÇÃO

Designa-se por liofilização uma técnica especial de dessecação, que permite a secagem de um corpo após prévio congelamento, removendo-se a água congelada por sublimação à custa de vácuo,

de modo a dar-se, diretamente, a passagem do estado sólido ao gasoso, sem que em qualquer momento da operação o gelo formado retome o estado líquido (Prista et al, 1981).

Essa técnica permitiu a secagem de substâncias alteráveis, principalmente os produtos de origem biológica, os quais, na sua maioria, são destruídos em sua totalidade ou parcialmente quando submetidos aos processos convencionais de dessecação. Atualmente, a liofilização é considerada como sendo o método ideal para secagem de produtos termolábeis ou de natureza complexa em que figurem constituintes excessivamente sujeitos a alterações por vários agentes, como o calor, o oxigênio, a umidade e tantos outros. A congelação brusca e intensa a que as substâncias (ou corpo) a liofilizar são submetidas permite conservar todas as propriedades que possuíam no momento da congelação, mantendo-as integralmente uma vez secas por sublimação do gelo formado (Prista et al, 1981).

Um dos exemplos mais demonstrativo do que é possível conseguir-se com a liofilização quanto à preservação das características de um corpo é a conservação dos microorganismos. Neste caso, além de mantê-los vivos, após a reconstituição, são mantidos os mesmos caracteres morfológicos, culturais, bioquímicos e biológicos (Prista et al, 1981).

Podemos citar uma lista de vantagens em relação ao processo de liofilização (Prista et al, 1981):

1 – A baixa temperatura a que se opera evita qualquer alteração química das substâncias decomponíveis pelo calor. Por isso, um produto seco por esta técnica mantém inalterável a sua composição química original, a sua atividade terapêutica e outras propriedades características. Se for acondicionado e armazenado convenientemente, poderá manter-se sem alteração durante um longo período.

2 - A perda de constituintes voláteis está reduzida ao mínimo.

3 – Os produtos liofilizados apresentam uma estrutura que contribui para sua rápida dissolução, assegurando-se, assim, a reprodução fiel do produto original uma vez posto o liofilizado em contato com a fase líquida primitiva.

4 – Durante a secagem o desenvolvimento de microrganismos e as reações enzimáticas são inibidas pelas baixas temperaturas a que se opera, o mesmo acontecendo nos produtos secos, pois seu teor de água mínimo impossibilita qualquer manifestação vital.

5 – A tendência de certos produtos coagularem quando dessecados por outras técnicas está reduzida quando utilizado este processo.

Com estas vantagens, não é de se estranhar, que os produtos submetidos a liofilização estejam aumentando a cada dia. Entre eles, podemos citar, por exemplo: certos antibióticos (penicilina), substâncias contendo proteínas termolábeis, bactérias, vírus, tecidos humanos para enxertos, alimentos, plasma sanguíneo, soros, vacinas, hormônios, etc.

OBJETIVO GERAL

Avaliar a eficácia e segurança do soro liofilizado heterólogo trivalente (antibotrópico-laquético-crotálico), produzido pelo Instituto Butantan.

OBJETIVOS ESPECÍFICOS

1. Avaliar a eficácia do soro antiofídico trivalente liofilizado produzido pelo Instituto Butantan, através de avaliação clínica laboratorial.
2. Descrever os possíveis eventos adversos ocorridos até 25 dias após a soroterapia.

MÉTODOS

Trata-se de um ensaio clínico prospectivo randomizado (Fletcher e col, 1996) com vítimas de ofidismo. O estudo será orientado para avaliar a eficácia e segurança da nova formulação de soro heterólogo produzido pelo Instituto Butantan. Os eventos de interesse são: (1) sinais e sintomas associados ao acidente; (2) sinais e sintomas temporalmente associados à soroterapia; e (3) alterações nos exames laboratoriais.

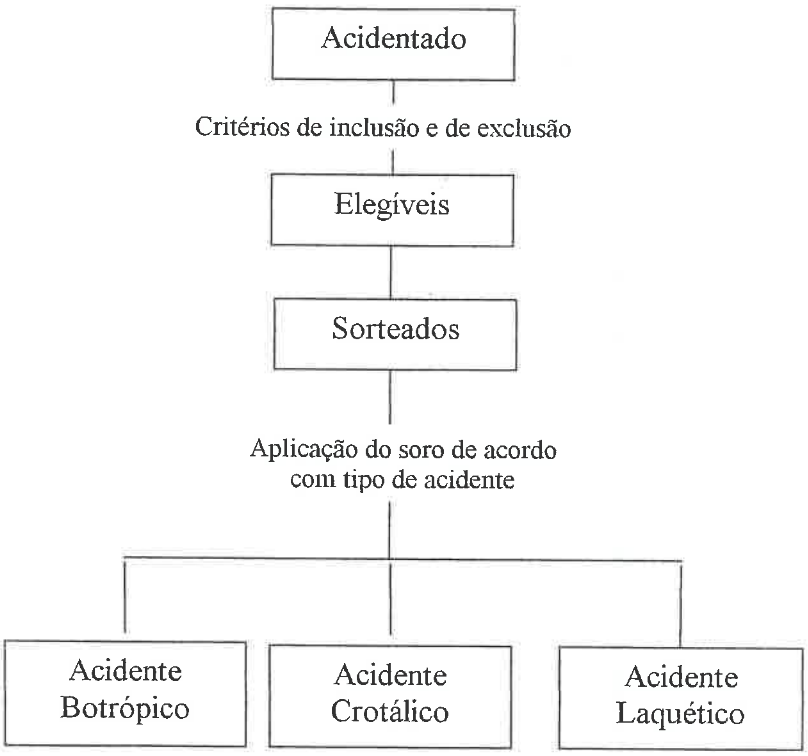

CRITÉRIOS DE ELEGIBILIDADE

1. Ter sido indicado para o uso do soro heterólogo por ocasião de acidente ofídico;
2. Ter idade entre 12 e 70 anos de idade;
3. Indivíduos do sexo feminino fora do estado de gravidez;
4. Não ser classificado na avaliação médica como caso grave;
5. Não ter usado soro antiofídico antes da chegada ao hospital que realiza o estudo;
6. Ler e assinar o Termo de Consentimento Livre e Esclarecido, o que poderá ser feito por um responsável no caso de impedimento da vítima.

CRITÉRIOS DE EXCLUSÃO

1. Gravidez no momento da aplicação da soroterapia;
2. Ter menos do que 12 e mais de 70 anos de idade;
3. Ser classificado na avaliação médica como caso grave;
4. Ter iniciado tratamento antiofídico antes da chegada ao hospital que realiza o estudo.

SOROTERAPIA

A soroterapia empregará o soro trivalente (antibotrópico-laquético-crotálico) na forma liofilizada produzido recentemente pelo Instituto Butantan em convênio com Instituto de Biologia do Exército. A distribuição, manipulação e aplicação do soro seguirão as recomendações do fabricante. Os procedimentos estão detalhados no Anexo III.

A soroterapia será realizada em uma Organização de Saúde por pessoal devidamente capacitado. Todo procedimento, incidentes e desvios de protocolo deverão ser registrados nos seus detalhes (Anexos IV, V, VI e VII) e comunicados aos coordenadores o mais breve possível. O estudo será suspenso caso ocorra reações graves e inesperadas, isto é, não descritos na literatura para a utilização dos soros comercializados.

SORO UTILIZADO NO ESTUDO

O soro antiofídico é uma solução que contém imunoglobulinas purificadas, obtidas a partir de plasma de eqüídeos hiperimunizados, com veneno da serpente a que se refere (Anexo X). Segue abaixo a formulação do soro a ser empregado no estudo:

O soro é apresentado em frascos-ampola contendo fração $F(ab^7)_2$ de imunoglobulinas, purificadas e liofilizadas, obtidas de plasma de eqüinos hiperimunizados com venenos de serpentes dos gêneros *Bothrops, Lachesis e Crotalus.* Acompanha o produto 05 ampolas de 20 ml de diluente para soro.

O soro tem a seguinte composição química:

1- fração $F(ab^7)_2$ de imunoglobulinas que neutralizam, no mínimo 100 mg, 60 mg e 30 mg dos venenos de referência de *Bothrops jararaca, Lachesis muta e Crotalus durissus terrificus*, respectivamente (soroneutralização em camundongo)

2- Fenol............................... 35mg (máximo)

3- Sacarose......................... 1g

4- Cloreto de Sódio............ 0,17g

Cada ampola de diluente contém 20ml de água purificada para injetáveis.

PLANO AMOSTRAL

Serão utilizados, inicialmente, no estudo piloto, 80 indivíduos vítimas de ofidismo, sendo distribuídos em três grupos conforme o tipo de acidente. O tipo de acidente será classificado de acordo com os seguintes gêneros de ofídios: *Bothrops*, *Crotalus*, e *Lachesis*.

A proporção de indivíduos em cada grupo será estabelecida pelo perfil epidemiológico da distribuição dos três principais gêneros de ofídios envolvidos nos acidentes em nosso país. Este perfil apresenta cerca de 90% para o botrópico, 8% para o crotálico e 1,5% para o laquético. Portanto a meta a ser alcançada será de 70 indivíduos classificados como acidente botrópico, 6 com acidente crotálico e 4 com acidente laquético, atingindo um subtotal de 40 acidentados para o uso do soro liofilizado e 40 para o uso do controle que serão os soros antiofídicos de apresentação líquida, antibotrópico-laquético e antibotrópico-crotálico, já produzidos pelo Instituto Butantan. Como o trabalho será realizado na Região Amazônica e a coordenação dos resultados centralizada no IBEx (Rio de Janeiro), caberá aos coordenadores informar aos participantes na medida em que estes objetivos numéricos forem atingidos. A finalidade é que seja empregado o menor número de voluntários possíveis a fim de comprovar a eficácia do soro trivalente liofilizado.

RECRUTAMENTO DE PARTICIPANTES

A seleção dos componentes da amostra será realizada pelo médico responsável pelo atendimento inicial que após avaliar a situação do acidentado e identificá-lo como possível elegível para o estudo, oferecerá a proposta de participação e posterior sorteio para o uso do soro líquido ou liofilizado. O concorde é efetivado após assinatura do Termo de Consentimento Livre e Esclarecido (Anexo VIII). Se o individuo for menor de idade, deverá ser preenchido um Termo de Consentimento Livre e Esclarecido Especial (Anexo IX) que deverá ser assinado pelo responsável legal pelo menor.

Será explicado ao paciente o objetivo do trabalho e sua metodologia, podendo o voluntário ou seu responsável (em caso do menor de idade) questionar qualquer etapa, ou desistir de participar a

qualquer momento. O termo será lido pelo médico, ou auxiliar de pesquisa, e após assinatura pelo voluntário ou responsável (em caso da menoridade do acidentado), se iniciará a entrevista (Anexo IV) e a soroterapia (Anexo III).

COLETA DE DADOS

Aos indivíduos que concordarem em participar do estudo, será aplicado um questionário (Anexo III) e preenchida um formulário de acompanhamento (Anexo V).

O questionário tem como objetivo obter informações da história pregressa do paciente. O questionário é dividido em duas seções: (1) dados gerais (nome completo do acidentado, filiação, idade, data do nascimento, endereço residencial completo e telefone, ocupação e endereço do trabalho); e (2) pré-soroterapia (antecedentes de uso de soro heterólogo e de manifestações alérgicas).

O formulário será preenchido pelo médico e seus auxiliares com dados da anamnese, exame físico, medicamentos recebidos durante a participação no estudo, eventos adversos e outras intercorrências até o 25° dia depois do início do tratamento. Os resultados dos exames laboratoriais (fotocópia legível) deverão ser encaminhados a Coordenação Central do Estudo (IBEx) logo após o 25° dia. A técnica utilizada para processamento de cada exame deverá ser informada com os documentos encaminhados e acima descritos.

A ocorrência de evento adverso grave deverá ser comunicada a coordenação (IBEx) o mais breve possível, menos de 24 horas do início da ocorrência, por comunicação telefônica direta. O objetivo é reconhecer o evento e se necessário paralisar o estudo até completa elucidação do evento.

TESTES LABORATORIAIS

Na admissão do paciente, antes do tratamento, deverá ser colhida uma amostra de sangue para realização dos exames laboratoriais previstos. As amostras de sangue serão processadas conforme rotina do laboratório de apoio da Unidade de Tratamento e todas serão identificadas com etiquetas auto-adesivas, deverá constar a metodologia empregada para realização de cada exame. Outras etiquetas, com o mesmo número, serão afixadas no questionário do participante e no cartão de identificação (Anexo VI). Nas amostras de sangue serão realizadas as seguintes análises séricas: hemograma completo, velocidade de hemossedimentação, alaninoaminotransferase (ALT), aspartatoaminotransferase (AST), fosfatase alcalina (FA), desidrogenase lática (LDH), creatinofosfoquinase (CK), uréia, creatinina, potássio, sódio, cálcio, bilirrubina total e frações, provas de coagulação sangüinea (tempo de coagulação, tempo de atividade da protrombina, tempo de tromboplastina parcial ativada, fibrinogênio

e detecção de veneno para ofídios pela técnica ELISA. A detecção de veneno será importante para identificar o gênero do ofídio causador do acidente e, avaliar a venenemia antes e após a soroterapia. As amostras de urina serão colhidas para realização do exame EAS (Elementos Anormais e Sedimentoscopia).

Os resultados dos testes laboratoriais e sua interpretação serão informados aos acidentados quando no retorno para as reavaliações. Os resultados dos exames e as fichas e anexos preenchidos serão enviados (correio, e-mail ou fax) para a Subdivisão de Pesquisa, Instituto de Biologia do Exército, Rua Francisco Manuel, Nr 102 – Benfica - Rio de Janeiro, RJ, CEP: 20911-270 ou dep.ibex@veloxmail.com.br ou silva.iran@ig.com.br ou ainda Tel/Fax 28605782 ou Tel. 38600442 ramal 228.

PLANO DE ANÁLISE DOS DADOS

Os dados do questionário, formulários e os resultados dos testes laboratoriais serão digitados duas vezes utilizando-se o EPI-INFO versão 6.04b (Dean *et al.*, 1994). O banco de dados será analisado no próprio EPI-INFO e os resultados exportados para a confecção dos gráficos no programa EXCEL.

Serão observadas as seguintes etapas de análise:

1° - Distribuição de freqüências das variáveis estudadas (quadro abaixo). Análise Multivariada.

- Acidente (botrópico, crotálico e laquético)
- Grau do acidente (grave, moderado, leve)
- Idade
- Local do acidente (braços, pernas, outros)
- Reações adversas
- Exames laboratoriais
- Exames clínicos
- Outros

2° - As variáveis serão submetidas à análise de tendência linear, com teste χ^2 para verificar a distribuição do desfecho nos diferentes grupos de exposição. A seguir, cada variável independente será organizada em dois grupos (dicotomizadas) e extraídos os Odds Ratio (OR) e os intervalos de confiança (IC).

CONSIDERAÇÕES ÉTICAS

Os benefícios da participação na pesquisa serão individuais e coletivos e resultarão da verificação da segurança na utilização do soro trivalente liofilizado. O soro apresenta melhores condições de conservação e, poderá ser de extremo benefício para tropas em operações em regiões distantes dos meios de conservação do produto e expostas a acidentes ofídicos. Em se tratando da população em geral residindo em localidades distantes e de difícil acesso, os benefícios individuais e coletivos serão os

mesmos que para os militares. Os benefícios individuais são os pertinentes à neutralização da peçonha evitando o risco de vida e possíveis seqüelas, como também disponibilizando tratamento eficaz apesar do desconhecimento do gênero do ofídio envolvido. Os inconvenientes se resumem àqueles associados a venopunção e aos possíveis efeitos adversos do soro heterólogo, já descritos na literatura e, em geral pouco observados na prática clínica.

A venopunção realizada por técnicos treinados é um procedimento bastante seguro. Os efeitos adversos do soro constituem-se de: reações anafiláticas, reações anafilactóides e reações pirogênicas, consideradas imediatas e doença do soro, considerada como reação tardia, que pode ocorrer de 5 a 24 dias da soroterapia. Todas estas reações foram profundamente estudadas e descritas com detalhes na prática médica, apresentam medidas de controle e atualmente não constituem fatores de risco impeditivos ao uso da soroterapia heteróloga, considerando que não há nenhuma outra modalidade terapêutica eficaz para o acidente ofídico.

As reações anafiláticas (hipersensibilidade tipo I) são reações mediadas pelas imunoglobulinas E, portanto passíveis de serem detectadas por meio de teste alérgico intradérmico. Atualmente não tem sido recomendado o teste, o Guia de Vigilância Epidemiológica da FUNASA (Ministério da Saúde) em vigor desde agosto de 2002, em seu Capítulo 5 que trata da "Vigilância Epidemiológica de Doenças e Agravos Específicos", mais especificamente no item 5.1 sobre "Acidentes por Animais Peçonhentos", através de uma nota prescreve o seguinte: "pela baixa capacidade em prever reações alérgicas, a prova intradérmica foi abolida da rotina, não sendo mais recomendada". Desta forma, com base no fato de que nas regiões onde não há sistema de refrigeração para conservação do soro líquido, há também uma grande dificuldade de transporte e atendimento do acidentado em tempo hábil, torna-se relevante o uso quase que imediato da soroterapia para evitar complicações do acidente ofídico. Sendo a aplicação do soro liofilizado, supervisionada pelo médico que tem condições de realizar uma prévia anamnese e atender as intercorrências reacionais.

As reações anafilactóides não são mediadas por anticorpos e independem de exposição prévia aos alérgenos ou antígenos do cavalo (pêlos, carne e soro). As medidas a serem tomadas nas reações anafiláticas e anafilactóides são as mesmas: interrupção imediata do gotejamento se o soro heterólogo estiver sendo administrado; aplicação subcutânea de 1 (um) ml de adrenalina solução aquosa na diluição 1:1.000, em caso de parada cardíaca aplicar pela via intravenosa ou intracardíaca; aplicação intramuscular ou intravenosa de uma ampola de prometazina; se houver broncoespasmo, aplicação de uma ampola de aminofilina diluída em 100ml soro glicosado 5% (gota-gota) e oxigenioterapia; hidrocortisona 500 mg intravenosa e diluída em 100 ml de soro glicosado 5%; se houver indicação será feita a entubação endotraqueal ou traqueostomia para manter uma via de acesso a oxigenioterapia. As reações pirogênicas são causadas pela interação do soro antipeçonhento, ou de endotoxinas bacterianas existentes no soro, com os macrófagos do indivíduo; manifesta-se inicialmente com arrepios de frio e posteriormente calafrios,

vindo após a febre; as medidas tomadas serão: a interrupção do gotejamento do soro e a aplicação intravenosa de 2 a 4 ml de dipirona.

A doença do soro é causada pela deposição de imunocomplexos formados entre o soro heterólogo e os anticorpos da classe IgG e IgM, desenvolvidos pelo próprio paciente, é uma reação do tipo III (classificação de Gell e Coombs) dependente da dose de antígeno inoculada no doente. As lesões tissulares, decorrentes da deposição de imunocomplexos, são evidenciadas pelas vasculites, glomerulonefrites e sinovites. Após a completa eliminação dos complexos, as lesões desaparecem rapidamente levando à perfeita recuperação dos tecidos. O primeiro sinal da doença é em geral um exantema pruriginoso, o qual pode ser urticariforme, maculopapular ou apenas eritematoso. Pode ser acompanhado de angioedema e inflamação no local da injeção. Além disso, o quadro clínico é acompanhado de febre, linfadenopatia, artralgias e mialgias. As articulações podem se apresentar edemaciadas, vermelhas, quentes e dolorosas. Pode ocorrer cefaléia e vômitos, os acometimentos cardíaco e renal não são freqüentes e pode ocorrer mononeurite, geralmente do plexo braquial, raramente ocorre polineurites, síndrome de Guillain-Barré ou meningoencefalite. As alterações hepáticas são transitórias e podem ocorrer elevações nas transaminases, discreta leucocitose com eosinofilia, aumento do VHS, proteinúria e hematúria, alterações eletrocardiográficas transitórias. Muito raramente pode ocorrer edema de glote com insuficiência respiratória, as neurites são em geral reversíveis e a recuperação total ocorre geralmente em 7 a 30 dias. O tratamento da doença do soro é conservador e sintomático, dependendo da manifestação presente, pode-se usar aspirina, dexclorofeniramina e corticosteróides. As reações adversas deverão ser registradas no Anexo VI.

Um Termo de Consentimento Livre e Esclarecido (Anexos VIII e IX) será solicitado do participante, enfatizando que (1) o consentimento solicitado é para o estudo; (2) que a participação acarreta mais benefícios do que riscos para a saúde; (3) que os resultados dos exames laboratoriais, bem como, todas as informações prestadas pelo participante serão tratadas como informações confidenciais e que ao estudo só interessam os dados consolidados sem a identificação individual; (4) que não serão realizados outros testes ou exames no material coletado, além daqueles previstos neste protocolo; (5) que todos os participantes serão informados dos resultados dos seus exames laboratoriais; (6) que a participação é voluntária e que os indivíduos são livres para retirar-se do estudo em qualquer fase, sem que isso implique em qualquer restrição. Será realizada palestra de esclarecimento nos hospitais envolvidos e, se possível, com as lideranças das comunidades locais antes do início da pesquisa. Um Termo de Consentimento Livre e Esclarecido especial será preenchido pelo responsável em caso do acidentado ser menor de idade.

CRONOGRAMA DA PESQUISA APÓS APROVAÇÃO PELO COMITÊ DE ÉTICA E PESQUISA (CEP)

ANO I

Atividade	Mês											
	1º	2º	3º	4º	5º	6º	7º	8º	9º	10º	11º	12º
Definição de funções e responsabilidades.	X	X	X									
Elaboração de questionário, cartões e etiquetas.				X								
Seleção e treinamento da equipe do trabalho de campo.				X	X							
Recrutamento de participantes, coleta de sangue.						X	X	X	X	X	X	X
Aplicação do soro heterólogo antibotrópico-laquético-crotálico.						X	X	X	X	X	X	X
Análise dos dados.						X	X	X	X	X	X	X
Preparação de relatórios.												X

ANO II

Atividade	Mês											
	1º	2º	3º	4º	5º	6º	7º	8º	9º	10º	11º	12º
Recrutamento de participantes, coleta de sangue.	X	X	X	X	X	X	X	X	X	X	X	X
Aplicação do soro heterólogo antibotrópico-laquético-crotálico.	X	X	X	X	X	X	X	X	X	X	X	X
Análise dos dados.	X	X	X	X	X	X	X	X	X	X	X	X
Preparação de relatórios.	X	X	X	X	X	X	X	X	X	X	X	X

ORÇAMENTO DA PESQUISA (ANEXO XI)

CUSTOS COM DESLOCAMENTOS E DIÁRIAS

A Coordenação Central do Projeto será no IBEx tendo o Maj Med Iran Mendonça da Silva como principal responsável, que viajará para a Região Amazônica com o objetivo de viabilizar a execução do Projeto e coordenar o seu desenvolvimento. A Coordenação Regional será realizada na Região Amazônica pelo Dr. Antônio Magela Tavares (FMTAM – Manaus) e Dra. Carmem Lúcia Tavares de Oliveira Machado (HGR – Boa Vista). Os exames laboratoriais para detecção do veneno e identificação do ofídio serão realizados pela Dra. Kátia Cristina Bárbaro (Laboratório de Imunopatologia – Instituto Butantan – São Paulo). O transporte do material biológico (soro dos acidentados e veneno dos ofídios) da Região Amazônica para São Paulo será realizado pela Coordenação Central do Projeto.

CUSTOS COM MATERIAL DE ESCRITÓRIO

Descrito no ANEXO XI, destina-se à execução do projeto em todas as fases e tanto na Coordenação Central como nas Coordenações Regionais.

CUSTOS COM MATERIAL PARA PADRONIZAÇÃO DO TESTE ELISA PARA DETECÇÃO DO VENENO

Materiais e reagentes necessários para a padronização do teste de ELISA para os venenos botrópico, crotálico e laquético (a serem providenciados pela Coordenação do Projeto – IBEx), descrito no ANEXO XI.

Venenos

30 mg de *Bothrops atrox*
30 mg de *Lachesis muta*
30 mg de *Crotalus durissus ruruima*

Soros (que serão utilizados na padronização do teste de ELISA)

1 ampola de soro antibotrópico
1 ampola de soro anticrotálico
1 ampola de soro antilaquético
50 amostras de soro humano de nativos da Região Amazônica

Reagentes SIGMA

P 8287 OPD 1 caixa 10 mg
A 6917 conjugado (peroxidase) anti-horse IgG 1 ml
A 6154 conjugado (peroxidase) anti-rabbit IgG 1 ml

Materiais Plásticos Descartáveis

1 caixa placa 96 wells Maxisorp para ELISA, Nunc, 439454
4 racks com ponteiras de 200 microlitros
600 ponteiras de 200 microlitros
200 ponteiras de 5000 microlitros
200 ponteiras de 1000 microlitros
200 ponteiras de 20 microlitros
200 tubos Eppendorf (1,5 ml) transparente
200 pipetas de 10 ml

Animais – coelhos, necessários para a obtenção do segundo anticorpo que será utilizado no teste ELISA.

Sais e Reagentes

Necessários para o preparo das soluções dos tampões utilizados no teste.

Adjuvantes

Necessários para a imunização dos animais para obtenção de anticoprpos

Material de escritório e informática

Necessário para o processamento dos resultados.

Facilities

Biotério e manutenção dos animais por 3 meses;
Lavador de placas de ELISA;
Leitor de placas de ELISA;
Estufa;
Balança;
Phmetro;
Freezer;
Geladeira;
Computador.

EXAMES LABORATORIAIS PARA DIAGNÓSTICO E ACOMPANHAMENTO CLÍNICO
(Serão realizados no acompanhamento do paciente nos seguintes períodos: pré-soroterapia e pós-soroterapia com 4 horas, 12 horas, 24 horas, 7 dias e 15 dias. O custo está detalhado no ANEXO XI)

Exames	Quantidade
Hemograma completo (eritrograma e avaliação de plaquetas)	1.000
Velocidade de Hemossedimentação (VHS)	1.000
Alaninoaminotransferase (ALT)	1.000
Aspartatoaminotransferase (AST)	1.000
Fosfatase alcalina (FA)	1.000
Desidrogenase lática (LDH)	1.000
Creatinofosfoquinase (CPK)	1.000
Fibrinogênio	1.000
Tempo de Tromboplastina Parcial Ativado (TTPA)	1.000
Tempo de atividade da Protrombina (TAP)	1.000
Tempo de Coagulação (TC)	1.000
Uréia	1.000
Creatinina	1.000
Potássio	1.000
Sódio	1.000
Cálcio	1.000
Bilirrubina total e frações	1.000
Sorologia pela técnica ELISA (*)	160
Sedimentoscopia (EAS)	1.000
**Total**	**18.160**

REFERÊNCIAS BIBLIOGRÁFICAS

1- Barravieira, B., Acidentes Ofídicos. Ricardo Veronesi. Tratado de Infectologia (Ed.) 8ª Edição. Editora Guanabara Koogan, 129:1577, 1999.

2- Barravieira, B., Aspectos Clínicos e Terapêuticos dos Acidentes por Animais Peçonhentos. EPUB – 1999.

3- Barravieira, B., Acidentes Ofídicos. Ricardo Veronesi (Ed.) 8ª Edição. Editora Guanabara Koogan, 129:1577, 1999.

4- Barravieira, B., *Aspectos Clínicos e Terapêuticos dos Acidentes por Animais Peçonhentos*. EPUB – 1999.

5- Brasil, 2000. *Manual de diagnóstico e Tratamento de Acidentes por Animais Peçonhentos*. Brasília, Ministério da Saúde. 131p.

6- Fan, H. W. & Cardoso,J. L. Clinical Toxicology of Snake-Bites in South América. **In** Meier, J. & White, J. eds *Clinical Toxicology of Animal Poisons* .Boca Raton, CRS Press, cap 31:667-688, 1995.

7- Fletcher, H. F, Fletcher, S. W. & Wagner, E. H. Epidemiologia clínica: elementos essenciais. Cap 7: 168-171. 1996.

8- Pardal, P.P.O., Rezende, M.B. & Dourado, H.V., Acidentes Ofídicos. Raimundo Nonato Queiroz de Leão I*n Doenças Infecciosas e Parasitárias – Enfoque Amazônico*- Editora Cejup- 54:805-811, 1994.

9- Pardal, P.P.O., Rezende, M.B. & Dourado, H.V., Acidentes Ofídicos. Raimundo Nonato Queiroz de Leão ***in*** Doenças Infecciosas e Parasitárias – Enfoque Amazônico- Editora Cejup- 54:805-811, 1994.

10- Pierini, S. V., Warrel, D. A., de Paulo, A. & Theakston, R. D. G. High incidence of bites and stings by snakes and other animals among rubber tappers and Amazonian Indians of the Juruá Valley, Acre State, Brazil. *Toxicon* 34 (2): 225-236, 1996.

11- Pierini, S. V., Warrel, D. A., de Paulo, A. & Theakston, R. D. G. High incidence of bites and stings by snakes and Revista do Hospital de Guarnição de Tabatinga- Qualidade Total em Saúde (HGuT) – Comando Militar da Amazônia - 12ª Região Militar – Exército Brasileiro – ano 1, Out2001, nº 1.

12- Prista, L. N, Alves, A. C., Morgado, R. M. R. Técnica farmacêutica e farmácia galénica. Fundação Calouste gulbenkian. Lisboa. Portugal. 1981. 8ª Edição. Volume 1.

Relatório sobre as condições de atendimento do Hospital de Guarnição de São Gabriel da Cachoeira (HGuSGC) – Comando Militar da Amazônia – 12ª Região Militar – Exército Brasileiro- Ministério da Defesa – Período de janeiro de 2000 a fevereiro de 2001.

13- Souza, A . R. B. & Muniz, E. G.. *Jararacas e Acidentes Ofídicos no Brasil* . Fundação de Medicina

Tropical, Manaus, AM. 2000, 34p.

14- Vital Brazil, O.. History of primordial of snake-bite accident serotherapy. *Memórias do Instituto Butantan*, 49(1) : 7-20, 1987.

15- http://www.funasa.gov.br/pub/GVE/GVE0501f.htm– Acidentes por animais peçonhentos – Vigilância Epidemiológica de Doenças e Agravos Específicos - Guia de Vigilância de Epidemiologia – Cap-5.

16- Cardoso J.L.C.; França F.O.S.F.; Fan, H. W.; Málaque C.M.S.; Hering S.E. & Cupo P.. Animais Peçonhentos no Brasil – Biologia, Clínica e Terapêutica, 1ª edição, Cap- 6,7 e 8:72-107, 2003

Rio de Janeiro, 15 de Setembro de 2003.

Anexo III

Protocolo de Tratamento (SOROTERAPIA)

Procedimento para Soroterapia.

As ampolas do soro antiofídico liofilizado podem ser conservadas, segundo o fabricante, a temperatura ambiente, devendo ser usado apenas após a adição de diluente. O conteúdo de frasco-ampola deve apresentar-se límpido e transparente. Não deve ser usado se houver turvação ou presença de precipitado. Deve ser usado logo após a reconstituição.

A via de administração recomendada é a ***endovenosa*** e o soro diluído deve ser infundido em torno de 45 minutos, sob estrita vigilância médica e da enfermagem. No caso do **maleato de dextroclorfeniramina**, a administração para este projeto será por via oral ***(VO).***

Visando proteger o paciente contra possíveis reações adversas, a administração do soro deve ser precedida pela aplicação dos seguintes medicamentos: antagonista dos receptores H2 da histamina , **cimetidina 300 mg por via endovenosa *(EV)***; corticoesteróide, **hidrocortisona 500 mg por via endovenosa *(EV)*** e antagonista dos receptores H1 da histamina, **maleato de dextroclorfeniramina 5 mg (xarope) por via oral *(VO).*** Os medicamentos deverão ser administrados 20 minutos antes da soroterapia antiofídica e, após adequada diluição (para o caso dos injetáveis). As dosagens acima são utilizadas para pacientes adultos, porém no caso de pacientes com baixo peso, poderá ser necessário o cálculo do medicamento por Kg/peso.

Quadro I – Classificação quanto à gravidade dos acidentes por ofídios peçonhentos.

Quadro Ia

Classificação Manifestações	Acidente causado por *Bothrops* (jararaca)
**Leve**	Edema e dor local discretos *(até 2 segmentos), manifestações sistêmicas** ausentes, tempo de coagulação normal ou alterado.
**Moderado**	Edema e dor local evidentes *(de 3 a 4 segmentos), manifestações sistêmicas** ausentes ou presentes, tempo de coagulação normal ou alterado.
**Grave**	Edema e dor local intensos *(de 5 segmentos), manifestações sistêmicas** evidentes, tempo de coagulação normal ou alterado.

* O membro picado é dividido em 5 segmentos: 1. pé/mão; 2. ½ distal da perna/antebraço; 3. ½ proximal da perna/antebraço; 4. ½ distal da coxa/antebraço; 5. ½ proximal da coxa/braço.

**Manifestações sistêmicas = hemorragia grave, choque e anúria.

Quadro Ib

Classificação Manifestações	Acidente causado por *Crotalus* (cascavel)
Leve	Fácies miastênica/visão turva ausentes ou tardias, mialgia ausente, urina vermelha ou marrom ausente, oligúria/anúria ausentes e tempo de coagulação norma ou alterado.
Moderado	Fácies miastênica/visão turva discreta ou evidente, mialgia discreta ou ausente, urina vermelha ou marrom pouco evidente ou ausente, oligúria/anúria ausentes e tempo de coagulação normal ou alterado.
Grave	Fácies miastênica/visão turva evidente, mialgia presente, urina vermelha ou marrom presente, oligúria/anúria presentes ou ausentes e tempo de coagulação normal ou alterado.

Brasil, Ministério da Saúde, 1998.

Quadro Ic

Classificação Manifestações	Acidente causado por *Lachesis* (surucucu)
Leve	Não considerado, todo acidente é classificado como moderado ou grave.
Moderado	Dor, edema, calor e rubor localizados; tempo de coagulação normal ou alterado; manifestação hemorrágica leve ou ausente.
Grave	Dor, edema, calor e rubor localizados; tempo de coagulação normal ou alterado, manifestação hemorrágica ausente, leve, moderada ou intensa, bradicardia, diarréia, hipotensão arterial e choque.

Quadro II – Classificação para tratamento dos acidentes ofídicos peçonhentos com indicação do número de frascos do soro liofilizado (para acidentes leves e moderados como previsto no projeto).

Quadro IIa

Classificação do Acidente	Tratamento do acidente causado por *Bothrops* (jararaca) (número de frascos)
Leve	2 frascos
Moderado	4 frascos

Quadro IIb

Classificação do Acidente	Tratamento do acidente causado por *Crotalus* (cascavel) (número de frascos)
Leve	2,5 frascos
Moderado	5 frascos

Quadro IIc

Classificação do Acidente	Tratamento do acidente causado por *Lachesis* (surucucu) (número de frascos)
Moderado	5 frascos

Obs.: o acidente laquético é classificado como moderado ou grave.

OBS1: A avaliação laboratorial seguirá o seguinte cronograma:

Distribuição Temporal	Coagulograma (inclui TAP e PTTa)	Fibrinogênio	Bioquímica	Hemograma e Plaquetas	Urina (EAS)
Admissão	X	X	X	X	X
4 horas	X	X	X	X	X
12 horas	X	X	X	X	X
24 horas	X	X	X	X	X
7 dias	X	X	X	X	X
15 dias	X	X	X	X	X

OBS2: Os resultados dos exames serão transcritos para a folha nº 2 deste anexo, para isto serão fornecidas 6 cópias destas folhas.

OBS3: Deverá ser aposto no início de cada folha nº 2, a data e a hora de cada colheita de material para o exame.

Anexo IV

Formulário para Entrevista (DADOS GERAIS)

1 - IDENTIFICAÇÃO

1.1 Nº PROTOCOLO:		1.2 DATA: ______/______/________
1.3 NOME:		1.4 SEXO: ( ) Masc ( ) Fem
1.5 DATA NASC: _____/_____/_____		1.6 NATURALIDADE:
1.7 END RES:		1.8 TEL RES:
1.9 BAIRRO:	1.10 CIDADE:	1.11 ESTADO:
1.12 END TRAB:		1.13 TEL TRAB:
1.14 OCUPAÇÃO:		
1.15 ESCOLARIDADE:<br>[ ] 1. Analfabeto 2. 1º Grau 3. 2º Grau 4. Superior 5. Ignorado<br>Especifique: [ ] 1. Completo 2. Incompleto		

2. OUTRAS INFORMAÇÕES

2.1 Preenchido ( ) antes ( ) durante ( ) depois da soroterapia

Responsável:	
Data:	Unidade:

MANUAL PARA APLICAÇÃO – ANEXO IV

O formulário deverá ser preenchido após a confirmação do paciente como voluntário para o trabalho. Os dados poderão ser coletados diretamente com o voluntário ou acompanhante, ou ainda, através do prontuário (ficha de entrada / cadastro, etc) do paciente.

O preenchimento segue os seguintes passos:

1º Passo: Preenchimento do campo "1 – IDENTIFICAÇÃO".

1- IDENTIFICAÇÃO DO PACIENTE: Preencher nº do protocolo do paciente na pesquisa, data de atendimento, seu nome completo,sexo, data de nascimento (DD/MM/AAAA), naturalidade, endereço residencial, bairro, cidade, estado, telefone da residência, endereço do trabalho, telefone do trabalho, ocupação e escolaridade.

Após preencher a identificação do paciente realizar a avaliação clínica.

É muito importante que não ocorre um risco maior para o paciente que está participando da pesquisa, logo o médico deve avaliar como e quando vai realizar a entrevista com o paciente (ou responsável).

O entrevistador deve preencher todas as lacunas do formulário.

2º Passo: Preenchimento do campo "2 – OUTRAS INFORMAÇÕES".

Informar o momento do preenchimento da ficha: antes, durante ou depois da soroterapia.

3º Passo: Identificação do responsável pelo preenchimento (nome completo), data do preenchimento ou coleta dos dados e unidade (local do trabalho).

4º Passo: Após preenchimento enviar uma cópia legível para o IBEx.

Anexo V

Ficha de Acompanhamento

1- IDENTIFICAÇÃO

1.1 Nº PROTOCOLO:	1.2 PRONTUÁRIO:
1.3 NOME:	1.4 DATA: _____/______/_______

2. AVALIAÇÃO CLÍNICA

2.1 Queixa Principal:
2.2 História do acidente:
2.2.a: Data do acidente:
2.2.b: Local de ocorrência do acidente:
2.2.c: Horário do acidente:
2.2.d: Identificação do ofídio:
2.2.e:Tamanho aproximado do ofídio (centímetros):
2.2.f: Região corporal picada pelo ofídio:
2.2.g. Descrição das alterações locais:
2.2.h: Descrição das alterações sistêmicas:
2.2.i: Outras informações:
2.3. História Patológica Pregressa:
2.3.a: Outros acidentes com ofídios? ( ) não ( ) sim Descrever (ofídio, data, tratamento, alergia, reações adversas) ____________________ ____________________
2.3.b: Uso de soro heterólogo anteriormente: ( ) sim ( ) não
2.3.c: Motivo do uso (2.3.b)/qual soro?
2.3.d: A quanto tempo?
2.3.e: Alergias (soro): ( ) não ( ) sim quais ? ____________________
2.3.f: Alergias (outras): ( ) não ( ) sim quais ? ____________________

3. EXAMES REALIZADOS E RESULTADOS

Data: / / . ( )Admissão ( )4 horas ( )12 horas ( )24 horas ( )7 dias ( )15 dias

3.1 Material colhido antes da soroterapia: ( ) urina ( ) sangue

3.2 Exames realizados antes da soroterapia:

3.2.a Urina (EAS)
pH :__________
Proteínas:__________
Hemoglobina:__________
Piócitos:__________
Hemácias:__________
Cilindros:__________

Cristais:__________
Pigmentos biliares:__________
Nitritos:__________
Corpos cetônicos:__________
Urobilinogênio:__________
Glicose:__________
Bactérias:__________

3.2.b Sangue
Hemograma Completo
Hemácias:__________
Hemoglobina:__________
Hematócrito:__________
Leucócitos totais:__________
Basófilos:__________
Eosinófilos:__________
Mielócitos:__________
Metamielócitos:__________
Bastonetes:__________
Segmentados:__________
Linfócitos:__________
Monócitos:__________
Promielócitos:__________
Contagem de plaquetas:__________

Provas de Função Hepática
Alaninoaminotransferase (ALT):__________
Aspartatoaminotransferase (AST):__________
Fosfatase alcalina (FA):__________
Desidrogenase láctica (DHL):__________
Bilirrubina total (Bt):__________
Bilirrubina direta (Bd):__________
Bilirrubina indireta (Bi):__________

Provas de Função Renal
Uréia:__________
Creatinina:__________
Potássio:__________
Sódio:__________

Provas de Coagulação Sangüínea
Tempo de Coagulação (TC):__________
Tempo de Atividade da Protrombina (TAP) :__________

Tempo de Tromboplastina Parcial Ativada (TTPA):
____
Fibrinogênio:__________

Outros exames:
____
____
____
____
____
____
____

Creatinoquinase (CK):__________
Cálcio:__________

Velocidade de hemossedimentação (VHS):
____

3.3 Sorologia pela técnica ELISA (material colhido antes e depois da soroterapia -1 hora após), para avaliar a venenemia – neutralização: 1ª colheita em / / . 2ª colheita em / / .

3.4 Exames realizados durante a soroterapia
3.4.a Urina (caso não realizado colheita antes da soroterapia): colhido em / / .
3.4.b Sangue: colhido em / / .
Descrever quais exames:__________

3.5 Exames realizados depois da soroterapia
3.5.a Urina: colhido em / / .
3.5.b Sangue: colhido em / / .
Descrever quais exames:__________

Responsável:

Data: | Unidade:

2.4. Manifestações Clínicas

( ) Dor ( ) Edema ( ) Eritema ( ) Bolhas

( ) Úlceras ( ) Abscesso ( ) Hemorragia local ( ) Gengivorragia

( ) Epistaxe ( ) Hemoptise ( ) Hemorragia digestiva ( ) Hematúria

( ) Hemorragia da pele ( ) Oligúria ( ) Anúria ( ) Náuseas

( ) Dor abdominal ( ) Vômitos ( ) Diarréia ( ) Lipotímia

( ) Visão turva ( ) Choque ( ) Ptose palpebral ( ) Insuficiência respiratória

( ) Diplopia ( ) Sialorréia ( ) Icterícia ( ) Paralisia dos membros superiores

( ) Disfagia ( ) Mialgias ( ) Oftalmoplegia ( ) Paralisia dos membros inferiores

( ) Outras

2.4a: Outras informações relevantes:

____

____

2.5. Exame Físico

2.5a: Ectoscopia:

____

____

____

2.5b: Aparelho Respiratório:

____

____

2.5c: Aparelho Cardiovascular:

____

____

2.5d: Aparelho Digestivo:

____

____

2.5e: Aparelho Geniturinário:

____

____

2.5f: Exame dos Membros:

____

____

2.6. Outras informações relevantes:

____

____

____

____

____

MANUAL PARA APLICAÇÃO

O formulário deverá ser preenchido pelo médico responsável pelo tratamento ou profissional de saúde indicado por ele. Os dados serão coletados dos pacientes voluntários.

Os dados serão registrados durante o tratamento até o 25 º dia após o início da soroterapia.

O preenchimento segue os seguintes passos:

__1º Passo:__ O paciente se enquadra nos quesitos de elegibilidade.

IMPORTANTE:

a) Critérios de elegibilidade: Ter sido indicado para o uso do soro heterólogo por ocasião de acidente ofídico; Ter idade entre 12 e 70 anos de idade; Indivíduos do sexo feminino fora do estado de gravidez; Não ser classificado na avaliação médica como caso grave; Não ter usado soro antiofídico antes da chegada ao hospital que realiza o estudo e Ler e assinar o Termo de Consentimento Livre e Esclarecido, o que poderá ser feito por um responsável no caso de impedimento da vítima.

b) **Critérios de exclusão:** Gravidez no momento da aplicação da soroterapia; Ter menos do que 12 e mais de 70 anos de idade; Ser classificado na avaliação médica como caso grave ou Ter iniciado tratamento antiofídico antes da chegada ao hospital que realiza o estudo.

__2º Passo:__ Oferecer a opção do soro trivalente explicitando as vantagens (três espécies) e informando sobre os possíveis eventos adversos (semelhantes ao utilizado na rotina de tratamento). Esclarecer possíveis dúvidas e perguntar se poderia participar do projeto. Caso a resposta seja negativa, agradecer e, continuar o tratamento de rotina. Sendo a resposta positiva, realizar sorteio e iniciar tratamento.

O paciente não deverá saber se está utilizando o soro liofilizado.

__3º Passo:__ Preenchimento da Ficha de Acompanhamento:

<u>1– IDENTIFICAÇÃO DO PACIENTE</u>: Preencher nº do protocolo do paciente na pesquisa, nº do prontuário na Unidade que está atendendo, seu nome completo e a data de entrada para o tratamento.

Após preencher a identificação do paciente realizar a avaliação clínica.

É muito importante que não ocorra um risco maior para o paciente que está participando da pesquisa, logo o médico deve avaliar como e quando vai realizar a entrevista com o paciente (ou responsável).

O entrevistador deve preencher todas as lacunas do formulário.

lŏÆéýDÓ▸┐?)ié

2- AVALIAÇÃO CLÍNICA:

4º Passo: item "2.1": Queixa principal: Descrever os sinais e sintomas apresentados pelo acidentado no momento do atendimento médico.

5º Passo: item "2.2": Relatar a história do acidente, para isso preencher os subitens:

2.2.a: Data do acidente: dia, mês, ano. Exemplo: **2/07/04** (dois de julho de dois mil e quatro).

2.2.b: Local de ocorrência do acidente: Descrever o local com suas características. Ex.: residência, mata, lavoura, perto de rios, pedras, etc.

2.2.c: Horário do acidente: hora aproximada (utilizar escala de "0" a 23 horas) . Exemplo: 8h/20h, 12h/0h.

2.2.d: Identificação do ofídio: quando possível identificar o ofídio, para isso perguntar ao acidentado ou acompanhante. Pode se utilizar a identificação local.

2.2.e: Tamanho aproximado do ofídio (centímetros): quando possível. Exemplo +/- 45 cm.

2.2.f: Região corporal picada pelo ofídio: Descrever por exemplo: região posterior da perna direita, região lateral do tornozelo esquerdo, etc.

2.2g: Descrição das alterações locais: Descrever os sinais da picada e alterações como: edema, sangramento local, etc.

2.2h: Descrição das alterações sistêmicas: Descrever os sinais e sintomas como: hematúria, sangramento gengival, alterações pressóricas, alterações sensoriais, etc.

2.2i: Outras informações: Espaço reservado para qualquer informação que o entrevistador ou médico ache relevante.

6º Passo: item "2.3" deverá ser descrito a história patológica pregressa, conforme os subitens descritos:

2.3.a: Outros acidentes com ofídios? ( ) não ( ) sim Descrever (ofídio, data, tratamento, alergia, reações adversas).

2.3.b: Uso de soro heterólogo anteriormente: ( ) sim ( ) não.

2.3.c: Motivo do uso (2.3.b)/qual soro?

2.3.d: Anotar a data ou período. Exemplos: *fevereiro de 2001, 1999, dois anos atrás, etc.*

2.3.e: Anotar as manifestações alérgicas, possivelmente relacionadas ao uso do soro.

2.3.f: Anotar outras alergias relatadas pelo paciente.

7º Passo: Transcrever os exames realizados no paciente. **Todos os exames listados são importantes para a pesquisa. Não esquecer de preencher a data da realização dos exames assim como assinalar o período de realização do exame na admissão e em relação ao início da soroterapia (no início da folha 2 deste anexo).**

8º Passo: Após preenchimento, enviar para o IBEx.

MINISTÉRIO DA DEFESA
EXÉRCITO BRASILEIRO
CML 1ª R M
INSTITUTO DE BIOLOGIA DO EXÉRCITO
IBEx(Lab. Microscopia Clin. e Bacteriol /1894)

¢YU§–°|Á♥– e¼ñ³±ù¥♦ₒv♠ºEU¶▓ÕC‼ S
ýBpËM▲6õ_Ì2 [illegible] |Þü/âà¡µÉ-9
└▪9‡+¹ ¨◂ãC3–¿ÓÏƒÉ %©Wg17©

Nº Protocolo: ______________

Nº Prontuário: ______________

FICHA DE INVESTIGAÇÃO DE EVENTOS ADVERSOS PÓS-SOROTERAPIA ANTIOFÍDICA LIOFILIZADA (BOTRÓPICO-LAQUÉTICO-CROTÁLICO)

IDENTIFICAÇÃO DO PACIENTE

1-NOME DO PACIENTE	2-DATA DO NASCIMENTO: ___/___/___
3-ENDEREÇO (Rua,Avenida,nº Aptº.)	
4-[illegible]AIRRO OU LOCALIDADE:	5- MUNICÍPIO: / 6-UF
7- [illegible]ELEFONE PARA CONTATO: ( )	8- PROFISSÃO:

DADOS COMPLEMENTARES DO CASO

[illegible] Nº DE FRASCOS ADMINISTRADOS: ______	10- VIA DE APLICAÇÃO: ______
11- DATA DA APLICAÇÃO: ___/___/___	12- LAB.PRODUTOR: IB-IBEx
1[illegible]- Nº DO LOTE:	14- VALIDADE:

EVENTOS ADVERSOS – REAÇÕES PRECOCES (24 horas) E TARDIAS

15 - MARCAR COM UM "x""

MANIFESTAÇÕES:	1º	2º	3º	4º	5º	6º	7º	8º	9º	10º	11º	12º	13º	14º	15º	16º	17º	18º	19º	20º	21º	22º	23º	24º	25º
[illegible]TICÁRIA																									
[illegible]URIDO																									
[illegible]BOR FACIAL																									
DISPNÉIA																									
SIBILOS																									
[illegible]SSE																									
F[illegible]BRE (indicar T ºC)																									
T[illegible]EMORES																									
NAUSEAS																									
[illegible]R ABDOMINAL																									
[illegible]GIOEDEMA																									
[illegible]EMA DE GLOTE																									
ARRITMIA CARDÍACA																									
H[illegible]POTENSÃO ARTERIAL																									
L[illegible]FOADENOMEGALIA																									
[illegible]LGIA																									
NEFRITE																									
M[illegible]ONONEURITE																									
[illegible]FALÉIA																									
[illegible]MITOS																									
[illegible]TROS (especificar *):																									
OUTROS (especificar *):																									
[illegible]TROS (especificar *):																									
[illegible]TROS (especificar *):																									

* Especificar: ____

1[illegible] - Observações: ____

ATENDIMENTO HOSPITALAR DO EVENTO ADVERSO

☐ 1- SIM 2- NÃO

18- DATA:
ENTRADA: ___/___/___ SAÍDA: ___/___/___

19- ☐ 1- EMERGÊNCIA 2- ENFERMARIA

HOSPITAL DE ATENDIMENTO:

EVOLUÇÃO DO CASO

21- ☐ ÓBITO 22- ☐ CURA SEM SEQÜELAS 23- ☐ IGNORADO 24- ☐ CURA COM SEQÜELAS

Continua verso

Continuação

SINAIS E SINTOMAS ANTERIORES À SOROTERAPIA (ÚLTIMAS 72 HORAS)		
25- FEBRE ☐ Tº C: ______<br>1-SIM 2-NÃO	26- OUTROS (especificar):	27- HÁ QUANTO TEMPO ?<br>______dias ______ horas _____ min.

ANTECEDENTES PESSOAIS

28- ALERGIAS (medicamentos,etc...) ?: ☐ SIM ☐ NÃO
Descrever: : ______________________________

29- IMUNODEFICIÊNCIAS (neoplasias, Aids, etc...):

30- USO CONCOMITANTE DE MEDICAMENTOS (corticóides, etc...):

31- APRESENTOU EVENTOS ADVERSOS EM SOROTERAPIA ANTERIOR ?: ☐ SIM ☐ NÃO

32 – ESPECIFICAR:
____

DADOS DO INVESTIGADOR

33- INVESTIGADOR:______________________________ 34- DATA: _____/_____/_____

____
ASSINATURA DO INVESTIGADOR/CARIMBO

Tel p/ contato:______________________________
E-mail: ______________________________

MANUAL PARA APLICAÇÃO – ANEXO VI

O f[illegible]mulário deverá ser preenchido pelo médico responsável pelo acompanhamento ou profissional de saúde indicado por [illegible]. Os dados serão coletados dos pacientes voluntários.

Os dados serão coletados durante o tratamento até o 25 º dia após o início da soroterapia.

O p[illegible]enchimento segue os seguintes passos:

[illegible] Passo: Preencher o quadro superior direito com o número de protocolo na pesquisa e número de prontuário do paciente.

[illegible] Passo: Preencher o módulo de Identificação do Paciente (itens 1 a 8).

[illegible] Passo: Preencher o módulo correspondente aos "Dados Complementares do Caso" (itens 9 a 14).
Item "9": anotar o número total de frascos de soro utilizados no tratamento.
Item "10": data do início do tratamento.

[illegible] Passo: Preencher o módulo "Eventos Adversos – Reações Precoces (24 horas) e Tardias" (itens 15 e 16).
Marcar com um "X" a interseção da coluna (dia) e linha (manifestações relatadas). O período de observação vai do 1º ao 25º dia de tratamento, aplicação do soro liofilizado.
Se [illegible]cessário relatar outra manifestação encontrada nos espaços "Especificar" e " Observações".

[illegible] Passo: Preencher o módulo "Atendimento Hospitalar do Evento Adverso" (itens 17 a 20).

6º Passo: Preencher o módulo "Evolução do Caso" (itens 21 a 24).
Ma[illegible]ar com um "X" como o caso evoluiu.

[illegible] Passo: Preencher o módulo "Sinais e Sintomas Anteriores à Soroterapia (últimas 72 horas)" (itens 25 a 27).

8º Passo: Preencher o módulo "Antecedentes Pessoais" (itens 28 a 32).

[illegible] Passo: Preencher o módulo "Dados do Investigador" (itens 33 e 34).

10º Passo: Após preenchimento encaminhar para o IBEx.

Anexo VII

Ficha de Acompanhamento para Coleta de Sangue

IBEx – IB – FMTAM

Nº do Protocolo (Pesquisa) ______________

Nome: ____

Idt nº : ____________________________

Data da entrada: ____/____/200 Responsável : _________________

1ª Coleta de sangue: ____/____/200 . Na admissão. Responsável : _________________

2ª Coleta de sangue: ____/____/200 . (__/__/__). 4 hs pós-soroterapia. Responsável:____________

3ª Coleta de sangue: ____/____/200 . (__/__/__).12 hs pós-soroterapia. Responsável : __________

4ª Coleta de sangue: ____/____/200 . (__/__/__).24 hs pós-soroterapia. Responsável : __________

5ª Coleta de sangue: ____/____/200 . (__/__/__).7 dias pós-soroterapia.Responsável : __________

6ª Coleta de sangue: ____/____/200 . (__/__/__).15 dias pós-soroterapia.Responsável:__________

MANUAL PARA APLICAÇÃO – ANEXO VII

Esta ficha deverá ser preenchida após a confirmação do paciente como voluntário para o trabalho. Nesta ficha serão informadas as datas das coletas de sangue para avaliação bioquímica e sorológica. O preenchimento segue os seguintes passos:

1º Passo: Anotar o número do protocolo de participação na pesquisa, o nome completo e o número de identidade.

2º Passo: Anotar no campo **"data de entrada"** a data que o paciente iniciou o tratamento (aplicação do soro antiofidico trivalente liofilizado).

3º Passo: Anotar a data AGENDADA para cada coleta de sangue.

4º Passo: Colocar entre parênteses a data REAL da coleta. Não esquecer de anotar o nome do responsável pela coleta.

5º Passo: Após preenchimento enviar uma cópia legível para o IBEx.

Anexo VIII

Termo de Consentimento Livre e Esclarecido (TCLE)
Instituto de Biologia do Exército (RJ) – Instituto Butantan (SP) – Fundação de Medicina Tropical do Amazonas

AVALIAÇÃO CLÍNICO-TERAPÊUTICA DO SORO ANTIOFÍDICO TRIVALENTE (BOTRÓPICO- LAQUÉTICO-CROTÁLICO) LIOFILIZADO

Este documento visa dar ao senhor informações e pedir sua participação neste trabalho, realizado em conjunto pelo Instituto de Biologia do Exército, Instituto Butantan e Fundação de Medicina Tropical, cujo objetivo é analisar a eficácia e a possível presença de eventos adversos ao tratamento com uma nova formulação do soro antiofídico.

A nova formulação do soro antiofídico consiste em uma forma liofilizada (em pó) que fornece maior estabilidade em ambientes quentes e é capaz de neutralizar três tipos de venenos (antibotrópico-laquético-crotálico), isto é, atua nas picadas das serpentes: jararaca, surucucu e cascavel.

Os testes em laboratório realizados com o novo soro apresentaram resultados similares aos soros já produzidos pelo Instituto Butantan.

A participação na pesquisa consiste em: (1) utilizar o soro liofilizado trivalente ou o soro líquido no tratamento; (2) realizar exames laboratoriais através da colheita de urina e da retirada de sangue venoso por venopunção; (3) responder as perguntas realizadas pelos pesquisadores.

Sua participação nesta pesquisa é muito importante, mas é uma escolha somente sua. O senhor pode se recusar a participar, sem que isso o prejudique no tratamento. Todas as informações sobre os participantes neste estudo são confidenciais. Não serão divulgados nomes dos participantes em nenhuma hipótese. Os resultados dos exames realizados serão disponibilizados ao senhor, permanecendo uma cópia com o pesquisador para análise.

Declaro estar ciente das informações deste Termo de Consentimento, entendendo que poderei pedir esclarecimentos a qualquer tempo. Declaro dar meu consentimento para a participação nesta pesquisa, estando ciente de que uma outra cópia deste termo permanecerá arquivada pelos organizadores da pesquisa.

Participante: ____

Local e data: ________________, _______/_________/ 200 .

____

Assinatura do participante

____

Assinatura do responsável pela pesquisa no local do Tratamento

Contato OMS / Hosp: ____________________________ **Tel:**__________________

Contatos (RJ): Maj Iran
Tel: 021 XX 3860-0442 ramal 228 ou 232 (Instituto de Biologia do Exército)

Anexo IX

Termo de Consentimento Livre e Esclarecido (TCLE) Especial
Instituto de Biologia do Exército (RJ) – Instituto Butantan (SP) – Fundação de Medicina Tropical do Amazonas

AVALIAÇÃO CLÍNICO-TERAPÊUTICA DO SORO ANTIOFÍDICO TRIVALENTE (BOTRÓPICO- LAQUÉTICO-CROTÁLICO) LIOFILIZADO

Este documento visa dar ao Senhor, informações e pedir participação do menor acidentado, através de sua autorização e do menor neste trabalho, realizado em conjunto pelo Instituto de Biologia do Exército, Instituto Butantan e Fundação de Medicina Tropical, cujo objetivo é analisar a eficácia e a possível presença de eventos adversos ao tratamento com uma nova formulação do soro antiofídico.

A nova formulação do soro antiofídico consiste em uma forma liofilizada (em pó) que fornece maior estabilidade em ambientes quentes e é capaz de neutralizar três tipos de venenos (antibotrópico-laquético-crotálico), isto é, atua nas picadas das serpentes: jararaca, surucucu e cascavel.

Os testes em laboratório realizados com o novo soro apresentaram resultados similares aos soros já produzidos pelo Instituto Butantan.

A participação na pesquisa consiste em: (1) utilizar o soro liofilizado trivalente ou o soro líquido no tratamento; (2) realizar exames laboratoriais através da colheita de urina e da retirada de sangue venoso por venopunção; (3) responder as perguntas realizadas pelos pesquisadores.

Sua participação e do acidentado nesta pesquisa é muito importante, mas é uma escolha somente de ambos. Os senhores podem se recusar a participar, sem que isso prejudique o tratamento. Todas as informações sobre os participantes neste estudo são confidenciais. Não serão divulgados nomes dos participantes em nenhuma hipótese. Os resultados exames realizados serão disponibilizados ao senhor e ao acidentado, permanecendo uma cópia com o pesquisador para análise.

Declaro estar ciente das informações deste Termo de Consentimento, entendendo que poderei pedir esclarecimentos a qualquer tempo. Declaro dar meu consentimento para a participação do menor nesta pesquisa, estando ciente de que uma outra cópia deste termo permanecerá arquivada pelos organizadores da pesquisa.

Participante: ____

Local e data: ____________________, ________/__________/ 200 .

____

Assinatura do responsável pelo acidentado

____

Assinatura do responsável pela pesquisa no local do Tratamento

Contato OMS / Hosp: ______________________________ **Tel:** ________________

Contatos (RJ): Maj Méd Iran
Tel: 021 XX 3860-0442 ramal 228 (Instituto de Biologia do Exército)

AVALIAÇÃO CLÍNICO-TERAPÊUTICA

SORO ANTIOFÍDICO TRIVALENTE LIOFILIZADO

FORMULÁRIOS E MANUAIS DE APLICAÇÃO

PROTOCOLO Nº

Anexo IV

Formulário para Entrevista (DADOS GERAIS)

1- IDENTIFICAÇÃO

1.1 Nº PROTOCOLO:		1.2 DATA: ______/______/________
1.3 NOME:		1.4 SEXO: ( ) Masc ( ) Fem
1.5 DATA NASC: _____/_____/_____		1.6 NATURALIDADE:
1.7 END RES:		1.8 TEL RES:
1.9 BAIRRO:	1.10 CIDADE:	1.11 ESTADO:
1.12 END TRAB:		1.13 TEL TRAB:
1.14 OCUPAÇÃO:		
1.15 ESCOLARIDADE: [ ] 1. Analfabeto 2. 1° Grau 3. 2° Grau 4. Superior 5. Ignorado<br>Especifique: [ ] 1. Completo 2. Incompleto		

2. OUTRAS INFORMAÇÕES

3.1 Preenchido ( ) antes ( ) durante ( ) depois da soroterapia

Responsável:	
Data:	Unidade:

MANUAL PARA APLICAÇÃO – ANEXO IV

O formulário deverá ser preenchido após a confirmação do paciente como voluntário para o trabalho. Os dados poderão ser coletados diretamente com o voluntário ou acompanhante, ou ainda, através do prontuário (ficha de entrada / cadastro, etc) do paciente.

O preenchimento segue os seguintes passos:

1º Passo: Preenchimento do campo "1 – IDENTIFICAÇÃO".

1- IDENTIFICAÇÃO DO PACIENTE: Preencher nº do protocolo do paciente na pesquisa, data de atendimento, seu nome completo,sexo, data de nascimento (DD/MM/AAAA), naturalidade, endereço residencial, bairro, cidade, estado, telefone da residência, endereço do trabalho, telefone do trabalho, ocupação e escolaridade.

Após preencher a identificação do paciente realizar a avaliação clínica.

É muito importante que não ocorre um risco maior para o paciente que está participando da pesquisa, logo o médico deve avaliar como e quando vai realizar a entrevista com o paciente (ou responsável).

O entrevistador deve preencher todas as lacunas do formulário.

2º Passo: Preenchimento do campo "2 – OUTRAS INFORMAÇÕES".

Informar o momento do preenchimento da ficha: antes, durante ou depois da soroterapia.

3º Passo: Identificação do responsável pelo preenchimento (nome completo), data do preenchimento ou coleta dos dados e unidade (local do trabalho).

4º Passo: Após preenchimento enviar uma cópia legível para o IBEx.

Anexo III

Protocolo de Tratamento (SOROTERAPIA)

Procedimento para Soroterapia.

As ampolas do soro antiofídico liofilizado podem ser conservadas, segundo o fabricante, a temperatura ambiente, devendo ser usado apenas após a adição de diluente. O conteúdo de frasco-ampola deve apresentar-se límpido e transparente. Não deve ser usado se houver turvação ou presença de precipitado. Deve ser usado logo após a reconstituição.

A via de administração recomendada é a ***endovenosa (EV)*** e o soro diluído (no diluente próprio e depois em 400 ml de SF 0,9% ou SG5%) deve ser infundido em torno de 45 minutos, sob estrita vigilância médica e da enfermagem.

Visando proteger o paciente contra possíveis reações adversas, a administração do soro deve ser precedida pela aplicação ***endovenosa (EV)*** dos seguintes medicamentos: antagonista dos receptores H1 da histamina, **maleato de dextroclorfeniramina 5 mg para adultos (0,08 mg/kg para uso pediátrico)**; antagonista dos receptores H2 da histamina, **cimetidina 300 mg para adultos (10 mg/kg para uso pediátrico)**; corticoesteróide, **hidrocortisona 500 mg (10 mg/kg).** Os medicamentos deverão ser aplicados 20 minutos antes da soroterapia antiofídica e após adequada diluição. As dosagens acima são utilizadas para pacientes adultos, porém no caso de pacientes com baixo peso, poderá ser necessário o cálculo do medicamento por Kg/peso.

Quadro I – Classificação quanto à gravidade dos acidentes por ofídios peçonhentos.

Quadro Ia

Classificação Manifestações	Acidente causado por *Bothrops* (jararaca)
Leve	Edema e dor local discretos *(até 2 segmentos), manifestações sistêmicas** ausentes, tempo de coagulação normal ou alterado.
Moderado	Edema e dor local evidentes *(de 3 a 4 segmentos), manifestações sistêmicas** ausentes ou presentes, tempo de coagulação normal ou alterado.
Grave	Edema e dor local intensos *(de 5 segmentos), manifestações sistêmicas** evidentes, tempo de coagulação normal ou alterado.

* O membro picado é dividido em 5 segmentos: 1. pé/mão; 2. ½ distal da perna/antebraço; 3. ½ proximal da perna/antebraço; 4. ½ distal da coxa/antebraço; 5. ½ proximal da coxa/braço.

**Manifestações sistêmicas = hemorragia grave, choque e anúria.

Quadro Ib

Classificação Manifestações	**Acidente causado por *Crotalus* (cascavel)**
Leve	Fácies miastênica/visão turva ausentes ou tardias, mialgia ausente, urina vermelha ou marrom ausente, oligúria/anúria ausentes e tempo de coagulação norma ou alterado.
Moderado	Fácies miastênica/visão turva discreta ou evidente, mialgia discreta ou ausente, urina vermelha ou marrom pouco evidente ou ausente, oligúria/anúria ausentes e tempo de coagulação normal ou alterado.
Grave	Fácies miastênica/visão turva evidente, mialgia presente, urina vermelha ou marrom presente, oligúria/anúria presentes ou ausentes e tempo de coagulação normal ou alterado.

Brasil, Ministério da Saúde, 1998.

Quadro Ic

Classificação Manifestações	**Acidente causado por *Lachesis* (surucucu)**
Leve	Não considerado, todo acidente é classificado como moderado ou grave.
Moderado	Dor, edema, calor e rubor localizados; tempo de coagulação normal ou alterado; manifestação hemorrágica leve ou ausente.
Grave	Dor, edema, calor e rubor localizados; tempo de coagulação normal ou alterado, manifestação hemorrágica ausente, leve, moderada ou intensa, bradicardia, diarréia, hipotensão arterial e choque.

Quadro II – Classificação para tratamento dos acidentes ofídicos peçonhentos com indicação do número de frascos do soro liofilizado (para acidentes leves e moderados como previsto no projeto).

Quadro IIa

Classificação do Acidente	Tratamento do acidente causado por *Bothrops* (jararaca) (número de frascos)
Leve	1 a 2 frascos
Moderado	2 a 4 frascos

Quadro IIb

Classificação do Acidente	Tratamento do acidente causado por *Crotalus* (cascavel) (número de frascos)
Leve	2,5 frascos
Moderado	5 frascos

Quadro IIc

Classificação do Acidente	Tratamento do acidente causado por *Lachesis* (surucucu) (número de frascos)
Moderado	5 frascos

Obs.: o acidente laquético é classificado como moderado ou grave.

Anexo V

Ficha de Acompanhamento

1- IDENTIFICAÇÃO

1.1 Nº PROTOCOLO:	1.2 PRONTUÁRIO:
1.3 NOME:	1.4 DATA: _____/______/_______

2. AVALIAÇÃO CLÍNICA

2.1 Queixa Principal:
2.2 História do acidente:
2.2.a: Data do acidente:
2.2.b: Local de ocorrência do acidente:
2.2.c: Horário do acidente:
2.2.d: Identificação do ofídio:
2.2.e:Tamanho aproximado do ofídio (centímetros):
2.2.f: Região corporal picada pelo ofídio:
2.2.g. Descrição das alterações locais:
2.2.h: Descrição das alterações sistêmicas:
2.2.i: Outras informações:
2.3. História Patológica Pregressa:
2.3.a: Outros acidentes com ofídios? ( ) não ( ) sim Descrever (ofídio, data, tratamento, alergia, reações adversas) ____________ ____________
2.3.b: Uso de soro heterólogo anteriormente: ( ) sim ( ) não
2.3.c: Motivo do uso (2.3.b)/qual soro?
2.3.d: A quanto tempo?
2.3.e: Alergias (soro): ( ) não ( ) sim quais ? ____________
2.3.f: Alergias (outras): ( ) não ( ) sim quais ? ____________

3. EXAMES REALIZADOS E RESULTADOS

Data: / / . ( )Admissão ( )4 horas ( )12 horas ( )24 horas ( )7 dias ( )15 dias

3.1 Material colhido antes da soroterapia: ( ) urina ( ) sangue

3.2 Exames realizados antes da soroterapia:

3.2.a <u>Urina (EAS)</u>
pH :______
Proteínas:______
Hemoglobina:______
Piócitos:______
Hemácias:______
Cilindros:______
Cristais:______
Pigmentos biliares:______
Nitritos:______
Corpos cetônicos:______
Urobilinogênio:______
Glicose:______
Bactérias:______

3.2.b Sangue
<u>Hemograma Completo</u>
Hemácias:______
Hemoglobina:______
Hematócrito:______
Leucócitos totais:______
Basófilos:______
Eosinófilos:______
Mielócitos:______
Metamielócitos:______
Bastonetes:______
Segmentados:______
Linfócitos:______
Monócitos:______
Promielócitos:______
Contagem de plaquetas:______

<u>Provas de Função Hepática</u>
Alaninoaminotransferase (ALT):______
Aspartatoaminotransferase (AST):______
Fosfatase alcalina (FA):______
Desidrogenase láctica (DHL):______
Bilirrubina total (Bt):______
Bilirrubina direta (Bd):______
Bilirrubina indireta (Bi):______

<u>Provas de Função Renal</u>
Uréia:______
Creatinina:______
Potássio:______
Sódio:______

<u>Provas de Coagulação Sangüínea</u>
Tempo de Coagulação (TC):______
Tempo de Atividade da Protrombina (TAP) :______
Tempo de Tromboplastina Parcial Ativada (TTPA):______
Fibrinogênio:______

Outros exames:
______
______
______
______
______
______
______

Creatinoquinase (CK):______
Cálcio:______

Velocidade de hemossedimentação (VHS):______

3.3 Sorologia pela técnica ELISA (material colhido antes e depois da soroterapia -1 hora após), para avaliar a venenemia – neutralização: 1ª colheita em / / . 2ª colheita em / / .

3.4 Exames realizados durante a soroterapia
3.4.a Urina (caso não realizado colheita antes da soroterapia): colhido em / / .
3.4.b Sangue: colhido em / / .
Descrever quais exames:______

3.5 Exames realizados depois da soroterapia
3.5.a Urina: colhido em / / .
3.5.b Sangue: colhido em / / .
Descrever quais exames:______

Responsável:

Data: | Unidade:

2.4. Manifestações Clínicas

( ) Dor	( ) Edema	( ) Eritema	( ) Bolhas
( ) Úlceras	( ) Abscesso	( ) Hemorragia local	( ) Gengivorragia
( ) Epistaxe	( ) Hemoptise	( ) Hemorragia digestiva	( ) Hematúria
( ) Hemorragia da pele	( ) Oligúria	( ) Anúria	( ) Náuseas
( ) Dor abdominal	( ) Vômitos	( ) Diarréia	( ) Lipotímia
( ) Visão turva	( ) Choque	( ) Ptose palpebral	( ) Insuficiência respiratória
( ) Diplopia	( ) Sialorréia	( ) Icterícia	( ) Paralisia dos membros superiores
( ) Disfagia	( ) Mialgias	( ) Oftalmoplegia	( ) Paralisia dos membros inferiores
( ) Outras			

2.4a: Outras informações relevantes:

____

____

2.5. Exame Físico

2.5a: Ectoscopia:

____

____

____

2.5b: Aparelho Respiratório:

____

____

2.5c: Aparelho Cardiovascular:

____

____

2.5d: Aparelho Digestivo:

____

____

2.5e: Aparelho Geniturinário:

____

____

2.5f: Exame dos Membros:

____

____

2.6. Outras informações relevantes:

____

____

____

____

____

OBS1: A avaliação laboratorial seguirá o seguinte cronograma:

Distribuição Temporal	Coagulograma (inclui TAP e PTTa)	Fibrinogênio	Bioquímica	Hemograma e Plaquetas	Urina (EAS)
Admissão	X	X	X	X	X
4 horas	X	X	X	X	X
12 horas	X	X	X	X	X
24 horas	X	X	X	X	X
7 dias			X	X	X
15 dias			X	X	X

OBS2: Os resultados dos exames serão transcritos para a folha nº 2 deste anexo, para isto serão fornecidas 6 cópias destas folhas.

OBS3: Deverá ser aposto no início de cada folha nº 2, a data e a hora de cada colheita de material para o exame.

MANUAL PARA APLICAÇÃO

O formulário deverá ser preenchido pelo médico responsável pelo tratamento ou profissional de saúde indicado por ele. Os dados serão coletados dos pacientes voluntários.

Os dados serão registrados durante o tratamento até o 25 º dia após o início da soroterapia.

O preenchimento segue os seguintes passos:

1º Passo: O paciente se enquadra nos quesitos de elegibilidade.

IMPORTANTE:

a) Critérios de elegibilidade: Ter sido indicado para o uso do soro heterólogo por ocasião de acidente ofídico; Ter idade entre 12 e 70 anos de idade; Indivíduos do sexo feminino fora do estado de gravidez; Não ser classificado na avaliação médica como caso grave; Não ter usado soro antiofídico antes da chegada ao hospital que realiza o estudo e Ler e assinar o Termo de Consentimento Livre e Esclarecido, o que poderá ser feito por um responsável no caso de impedimento da vítima.

b) **Critérios de exclusão:** Gravidez no momento da aplicação da soroterapia; Ter menos do que 12 e mais de 70 anos de idade; Ser classificado na avaliação médica como caso grave ou Ter iniciado tratamento antiofídico antes da chegada ao hospital que realiza o estudo.

2º Passo: Oferecer a opção do soro trivalente explicitando as vantagens (três espécies) e informando sobre os possíveis eventos adversos (semelhantes ao utilizado na rotina de tratamento). Esclarecer possíveis dúvidas e perguntar se poderia participar do projeto. Caso a resposta seja negativa, agradecer e, continuar o tratamento de rotina. Sendo a resposta positiva, realizar sorteio e iniciar tratamento.

O paciente não deverá saber se está utilizando o soro liofilizado.

3º Passo: Preenchimento da Ficha de Acompanhamento:

1– IDENTIFICAÇÃO DO PACIENTE: Preencher nº do protocolo do paciente na pesquisa, nº do prontuário na Unidade que está atendendo, seu nome completo e a data de entrada para o tratamento.

Após preencher a identificação do paciente realizar a avaliação clínica.

É muito importante que não ocorra um risco maior para o paciente que está participando da pesquisa, logo o médico deve avaliar como e quando vai realizar a entrevista com o paciente (ou responsável).

O entrevistador deve preencher todas as lacunas do formulário.

2– AVALIAÇÃO CLÍNICA:

4º Passo: item "2.1": Queixa principal: Descrever os sinais e sintomas apresentados pelo acidentado no momento do atendimento médico.

5º Passo: item "2.2": Relatar a história do acidente, para isso preencher os subitens:

2.2.a: Data do acidente: dia, mês, ano. Exemplo: **2/07/04** (dois de julho de dois mil e quatro).

2.2.b: Local de ocorrência do acidente: Descrever o local com suas características. Ex.: residência, mata, lavoura, perto de rios, pedras, etc.

2.2.c: Horário do acidente: hora aproximada (utilizar escala de "0" a 23 horas) . Exemplo: 8h/20h, 12h/0h.

2.2.d: Identificação do ofídio: quando possível identificar o ofídio, para isso perguntar ao acidentado ou acompanhante. Pode se utilizar a identificação local.

2.2.e: Tamanho aproximado do ofídio (centímetros): quando possível. Exemplo +/- 45 cm.

2.2.f: Região corporal picada pelo ofídio: Descrever por exemplo: região posterior da perna direita, região lateral do tornozelo esquerdo, etc.

2.2g: Descrição das alterações locais: Descrever os sinais da picada e alterações como: edema, sangramento local, etc.

2.2h: Descrição das alterações sistêmicas: Descrever os sinais e sintomas como: hematúria, sangramento gengival, alterações pressóricas, alterações sensoriais, etc.

2.2i: Outras informações: Espaço reservado para qualquer informação que o entrevistador ou médico ache relevante.

6º Passo: item "2.3" deverá ser descrito a história patológica pregressa, conforme os subitens descritos:

2.3.a: Outros acidentes com ofídios? ( ) não ( ) sim Descrever (ofídio, data, tratamento, alergia, reações adversas).

2.3.b: Uso de soro heterólogo anteriormente: ( ) sim ( ) não.

2.3.c: Motivo do uso (2.3.b)/qual soro?

2.3.d: Anotar a data ou período. Exemplos: *fevereiro de 2001, 1999, dois anos atrás, etc.*

2.3.e: Anotar as manifestações alérgicas, possivelmente relacionadas ao uso do soro.

2.3.f: Anotar outras alergias relatadas pelo paciente.

7º Passo: Transcrever os exames realizados no paciente. **Todos os exames listados são importantes para a pesquisa. Não esquecer de preencher a data da realização dos exames assim como assinalar o período de realização do exame na admissão e em relação ao início da soroterapia (no início da folha 2 deste anexo).**

8º Passo: Após preenchimento, enviar para o IBEx.

Anexo VIII

Termo de Consentimento Livre e Esclarecido (TCLE)
Instituto de Biologia do Exército (RJ) – Instituto Butantan (SP) – Fundação de Medicina Tropical do Amazonas
AVALIAÇÃO CLÍNICO-TERAPÊUTICA DO SORO ANTIOFÍDICO TRIVALENTE (BOTRÓPICO- LAQUÉTICO-CROTÁLICO) LIOFILIZADO

Este documento visa dar ao senhor informações e pedir sua participação neste trabalho, realizado em conjunto pelo Instituto de Biologia do Exército, Instituto Butantan e Fundação de Medicina Tropical, cujo objetivo é analisar a eficácia e a possível presença de eventos adversos ao tratamento com uma nova formulação do soro antiofídico.

A nova formulação do soro antiofídico consiste em uma forma liofilizada (em pó) que fornece maior estabilidade em ambientes quentes e é capaz de neutralizar três tipos de venenos (antibotrópico-laquético-crotálico), isto é, atua nas picadas das serpentes: jararaca, surucucu e cascavel.

Os testes em laboratório realizados com o novo soro apresentaram resultados similares aos soros já produzidos pelo Instituto Butantan.

A participação na pesquisa consiste em: (1) utilizar o soro liofilizado trivalente ou o soro líquido no tratamento; (2) realizar exames laboratoriais através da colheita de urina e da retirada de sangue venoso por venopunção; (3) responder as perguntas realizadas pelos pesquisadores.

Sua participação nesta pesquisa é muito importante, mas é uma escolha somente sua. O senhor pode se recusar a participar, sem que isso o prejudique no tratamento. Todas as informações sobre os participantes neste estudo são confidenciais. Não serão divulgados nomes dos participantes em nenhuma hipótese. Os resultados dos exames realizados serão disponibilizados ao senhor, permanecendo uma cópia com o pesquisador para análise.

Declaro estar ciente das informações deste Termo de Consentimento, entendendo que poderei pedir esclarecimentos a qualquer tempo. Declaro dar meu consentimento para a participação nesta pesquisa, estando ciente de que uma outra cópia deste termo permanecerá arquivada pelos organizadores da pesquisa.

Participante: ____

Local e data: ________________, ________/__________/ 200 .

____

Assinatura do participante

____

Assinatura do responsável pela pesquisa no local do Tratamento

Contato OMS / Hosp: ______________________________ Tel:__________________

Contatos (RJ): Maj Iran
Tel: 021 XX 3860-0442 ramal 228 ou 232 (Instituto de Biologia do Exército)

Anexo IX

Termo de Consentimento Livre e Esclarecido (TCLE) Especial
Instituto de Biologia do Exército (RJ) – Instituto Butantan (SP) – Fundação de Medicina Tropical do Amazonas

AVALIAÇÃO CLÍNICO-TERAPÊUTICA DO SORO ANTIOFÍDICO TRIVALENTE (BOTRÓPICO- LAQUÉTICO-CROTÁLICO) LIOFILIZADO

Este documento visa dar ao Senhor, informações e pedir participação do menor acidentado, através de sua autorização e do menor neste trabalho, realizado em conjunto pelo Instituto de Biologia do Exército, Instituto Butantan e Fundação de Medicina Tropical, cujo objetivo é analisar a eficácia e a possível presença de eventos adversos ao tratamento com uma nova formulação do soro antiofídico.

A nova formulação do soro antiofídico consiste em uma forma liofilizada (em pó) que fornece maior estabilidade em ambientes quentes e é capaz de neutralizar três tipos de venenos (antibotrópico-laquético-crotálico), isto é, atua nas picadas das serpentes: jararaca, surucucu e cascavel.

Os testes em laboratório realizados com o novo soro apresentaram resultados similares aos soros já produzidos pelo Instituto Butantan.

A participação na pesquisa consiste em: (1) utilizar o soro liofilizado trivalente ou o soro líquido no tratamento; (2) realizar exames laboratoriais através da colheita de urina e da retirada de sangue venoso por venopunção; (3) responder as perguntas realizadas pelos pesquisadores.

Sua participação e do acidentado nesta pesquisa é muito importante, mas é uma escolha somente de ambos. Os senhores podem se recusar a participar, sem que isso prejudique o tratamento. Todas as informações sobre os participantes neste estudo são confidenciais. Não serão divulgados nomes dos participantes em nenhuma hipótese. Os resultados exames realizados serão disponibilizados ao senhor e ao acidentado, permanecendo uma cópia com o pesquisador para análise.

Declaro estar ciente das informações deste Termo de Consentimento, entendendo que poderei pedir esclarecimentos a qualquer tempo. Declaro dar meu consentimento para a participação do menor nesta pesquisa, estando ciente de que uma outra cópia deste termo permanecerá arquivada pelos organizadores da pesquisa.

Participante: ____

Local e data: ____________________, ________/___________/ 200 .

____

Assinatura do responsável pelo acidentado

____

Assinatura do responsável pela pesquisa no local do Tratamento

Contato OMS / Hosp: ________________________________ Tel:________________

Contatos (RJ): Maj Méd Iran
Tel: 021 XX 3860-0442 ramal 228 (Instituto de Biologia do Exército)

AnexoVI

MINISTÉRIO DA DEFESA
EXÉRCITO BRASILEIRO
CML 1ª R M
INSTITUTO DE BIOLOGIA DO EXÉRCITO
IBEx(Lab. Microscopia Clín. e Bacteriol /1894)

Nº Protocolo: ____________

Nº Prontuário: ____________

FICHA DE INVESTIGAÇÃO DOS EVENTOS ADVERSOS PÓS-SOROTERAPIA ANTIOFÍDICA LIOFILIZADA (BOTRÓPICO-LAQUÉTICO-CROTÁLICO)

IDENTIFICAÇÃO DO PACIENTE

1-NOME DO PACIENTE

2-DATA DO NASCIMENTO: ____/____/____

3-ENDEREÇO (Rua,Avenida,nº Aptº.)

4-BAIRRO OU LOCALIDADE:

5- MUNICÍPIO:

6-UF

7- TELEFONE PARA CONTATO:
( )

8- PROFISSÃO:

DADOS COMPLEMENTARES DO CASO

9- Nº DE FRASCOS ADMINISTRADOS: ______

10- VIA DE APLICAÇÃO: ____________

11- DATA DA APLICAÇÃO: ____/____/____

12- LAB.PRODUTOR: IB-IBEx

13- Nº DO LOTE:

14- VALIDADE:

EVENTOS ADVERSOS – REAÇÕES PRECOCES (24 horas) E TARDIAS

15 - MARCAR COM UM "x""

MANIFESTAÇÕES:	1º	2º	3º	4º	5º	6º	7º	8º	9º	10º	11º	12º	13º	14º	15º	16º	17º	18º	19º	20º	21º	22º	23º	24º	2[illegible]
[illegible]RTICÁRIA																									
PRURIDO																									
RUBOR FACIAL																									
DISPNÉIA																									
SIBILOS																									
TOSSE																									
FEBRE (indicar T ºC)																									
TREMORES																									
NÁUSEAS																									
DOR ABDOMINAL																									
ANGIOEDEMA																									
EDEMA DE GLOTE																									
ARRITMIA CARDÍACA																									
HIPOTENSÃO ARTERIAL																									
LINFOADENOMEGALIA																									
MIALGIA																									
NEFRITE																									
[illegible]ONONEURITE																									
CEFALÉIA																									
VÔMITOS																									
OUTROS (especificar *):																									
OUTROS (especificar *):																									
OUTROS (especificar *):																									
OUTROS (especificar *):																									

* Especificar: ____________

16 - Observações: ____________

ATENDIMENTO HOSPITALAR DO EVENTO ADVERSO

17- ☐ 1- SIM 2- NÃO

18- DATA:
ENTRADA: ____/____/____ SAÍDA: ____/____/____

19- ☐ 1- EMERGÊNCIA 2- ENFERMARIA

20- HOSPITAL DE ATENDIMENTO:

EVOLUÇÃO DO CASO

21- ☐ ÓBITO 22- ☐ CURA SEM SEQÜELAS 23- ☐ IGNORADO 24- ☐ CURA COM SEQÜELAS

Continua verso

Continuação

SINAIS E SINTOMAS ANTERIORES À SOROTERAPIA (ÚLTIMAS 72 HORAS)

25- FEBRE [ ] T° C: ______ 1-SIM 2-NÃO	26- OUTROS (especificar):	27- HÁ QUANTO TEMPO ? ______dias ______ horas _____ min.

ANTECEDENTES PESSOAIS

28- ALERGIAS (medicamentos,etc...) ?: ☐ SIM ☐ NÃO

Descrever: :____

29- IMUNODEFICIÊNCIAS (neoplasias, Aids, etc...):

30- USO CONCOMITANTE DE MEDICAMENTOS (corticóides, etc...):

31- APRESENTOU EVENTOS ADVERSOS EM SOROTERAPIA ANTERIOR ?: ☐ SIM ☐ NÃO

32 – ESPECIFICAR:

____

DADOS DO INVESTIGADOR

33- INVESTIGADOR:____________________________ 34- DATA: ____/____/____

____

ASSINATURA DO INVESTIGADOR/CARIMBO

Tel p/ contato:______________________

E-mail: ______________________

MANUAL PARA APLICAÇÃO – ANEXO VI

O formulário deverá ser preenchido pelo médico responsável pelo acompanhamento ou profissional de saúde indicado por ele. Os dados serão coletados dos pacientes voluntários.

Os dados serão coletados durante o tratamento até o 25 º dia após o início da soroterapia.

O preenchimento segue os seguintes passos:

1º Passo: Preencher o quadro superior direito com o número de protocolo na pesquisa e número de prontuário do paciente.

2º Passo: Preencher o módulo de Identificação do Paciente (itens 1 a 8).

3º Passo: Preencher o módulo correspondente aos "Dados Complementares do Caso" (itens 9 a 14).
Item "9": anotar o número total de frascos de soro utilizados no tratamento.
Item "10": data do inicio do tratamento.

[illegible] Passo: Preencher o módulo "Eventos Adversos – Reações Precoces (24 horas) e Tardias" (itens 15 e 16).
Marcar com um "X" a interseção da coluna (dia) e linha (manifestações relatadas). O período de observação vai do 1º ao 25º dia de tratamento, aplicação do soro liofilizado.
Se necessário relatar outra manifestação encontrada nos espaços "Especificar" e " Observações".

[illegible] Passo: Preencher o módulo "Atendimento Hospitalar do Evento Adverso" (itens 17 a 20).

6º Passo: Preencher o módulo "Evolução do Caso" (itens 21 a 24).
Marcar com um "X" como o caso evoluiu.

[illegible] Passo: Preencher o módulo "Sinais e Sintomas Anteriores à Soroterapia (últimas 72 horas)" (itens 25 a 27).

8º Passo: Preencher o módulo "Antecedentes Pessoais" (itens 28 a 32).

[illegible] Passo: Preencher o módulo "Dados do Investigador" (itens 33 e 34).

10º Passo: Após preenchimento encaminhar para o IBEx.
