# Supplementary material for: Safety and efficacy of a freeze-dried trivalent antivenom for snakebites in the Brazilian Amazon: An open randomized controlled phase IIb clinical trial
Source: PLoS Negl Trop Dis. 2017 Nov 27;11(11):e0006068. doi: 10.1371/journal.pntd.0006068 (PMC5720814; doi:10.1371/journal.pntd.0006068)
Supplement: S1 File — (DOCX) [file pntd.0006068.s005.docx]

**Determination of anti-*Bothrops*, *Lachesis* and *Crotalus* antivenom potencies**

Determination of antivenom potencies used official standard methods recommended by the Brazilian Pharmacopeia [1].

**1. *Bothrops* antivenom potency assay**

The potency assay aims at determining the required neutralizing dose (Effective Dose 50%) to protect susceptible animals against the lethal effects of a fixed dose of a reference venom.

The reference venom is a homogeneous mixture of venoms that represent the geographical distribution of *B. jararaca* species. It should be lyophilized and maintained at -20°C. The venom is standardized by the determination of the 50% Lethal Dose (LD50).

**Determination of the LD50 of the venom:** Firstly, reconstitute the lyophilized venom at a determined concentration by volume with 0.85% saline (w/v). Make dilutions in geometric progression with the same diluent, using a constant dilution factor of not more than 1.5 and equalize the final volumes. Inoculate, by intraperitoneal route, a volume of 0.5 ml per mouse of each dilution in groups of at least 10 Swiss albino mice from 18 g to 22 g. Observe the animals up to 48 hours after inoculation and record the number of deaths at each dilution. Calculate the LD50 using a proven statistical method. In this study a Probit analysis was used [2]. The response range (percentage of deaths) should be between the highest and the lowest dilution used, forming the regression curve that must present a linear correlation. Confidence intervals should not be large, indicating better accuracy of the test, the smaller the limits. Express the result in micrograms of venom per 0.5 mL.

**Determination of antivenom potency:** Carry out progressive dilutions of the sample in saline at 0.85% (w/v) using a constant dilution factor of not more than 1.5, so that the final volume after mixing with the challenge dose is identical across all test tubes. Reconstitute and dilute the reference venom with 0.85% (w/v) saline solution and add constant volume to each tube so that each dose to be inoculated per animal contains 5xLD50. Homogenize and incubate the mixture at 37°C for 60 minutes. Inoculate intraperitoneally with a volume of 0.5 ml per mouse of each mixture in groups of at least eight Swiss albino mice of 18 g to 22 g. Observe the animals up to 48 hours after inoculation and record the number of live in each mixture. Calculate the 50% Effective Dose (ED50) in microliters, using a proven statistical method. In this study a Probit analysis was used [2]. The response range produced (percentage of survival) should be between the highest and the lowest dilution used, forming the regression curve that should present a linear correlation. Confidence intervals should not be large, indicating better accuracy of the test, the smaller the limits. Calculate the power in milligrams per milliliter, according to the expression:

**Antivenom Potency (mg/ml): (Tv-1/ED50) x venom DL50**

on what

Tv=number of LD50 used per mouse in the venom test dose.

Antivenom potency was expressed in milligrams of venom neutralized by 1 mL of the sample. It is accepted a 10% coefficient of variation.

The minimum *Bothrops* antivenom potency can vary up to 4.5 mg/mL.

**2. *Lachesis* antivenom potency assay**

The reference venom is a homogeneous mixture of venoms that represent the geographical distribution of *L. muta* species. It should be lyophilized and maintained at -20°C. The venom is standardized by the determination of the 50% Lethal Dose (LD50).

**Determination of the LD50 of the venom**: Use the same procedures presented for *Bothrops* venom.

**Determination of antivenom potency:** Use the same procedures presented for *Bothrops* antivenom.

The minimum *Lachesis* antivenom potency can vary up to 2.7 mg/mL.

**3. *Crotalus* antivenom potency assay**

The reference venom is a homogeneous mixture of venoms that represent the geographical distribution of *C. durissus* species. It should be lyophilized and maintained at -20°C. The venom is standardized by the determination of the 50% Lethal Dose (LD50).

**Determination of the LD50 of the venom**: Use the same procedures presented for *Bothrops* venom.

**Determination of antivenom potency:** Use the same procedures presented for *Bothrops* antivenom.

The minimum *Crotalus* antivenom potency can vary up to 1.35 mg/mL.

**Reference**

1. Brazilian Ministry of Health (2000) Farmacopeia Brasileira, 4th ed. Anvisa, Brasília.
2. Finney DJ (1971) Probit Analysis, 3rd ed. Cambridge University Press, Cambridge.
