# Supplementary material for: Safety and efficacy of a freeze-dried trivalent antivenom for snakebites in the Brazilian Amazon: An open randomized controlled phase IIb clinical trial
Source: PLoS Negl Trop Dis. 2017 Nov 27;11(11):e0006068. doi: 10.1371/journal.pntd.0006068 (PMC5720814; doi:10.1371/journal.pntd.0006068)
Supplement: S2 File — (DOCX) [file pntd.0006068.s006.docx]

**S2 File.** Baseline laboratorial features of the included patients and comparison of means between experimental groups in *Bothrops* snakebites.

| **Variable** | ***Bothrops* bites (Mean (SD)/n (%))** | | ***p*** |
| --- | --- | --- | --- |
|  | **Group A** | **Group B** |  |
| **Urea** (mg/dL) | 33.8 (11.1) | 32.5 (10.6) | 0.213 |
| **Creatinine** (mg/dL) | 1.1 (0.3) | 1.0 (0.2) | 0.602 |
| **K^+^** (mmol/L) | 4.5 (1.4) | 4.3 (1.3) | 0.677 |
| **Na^2+^**(mmol/L) | 140 (3.2) | 138 (2.0) | 0.741 |
| **Lactate dehydrogenase** (mg/dL) | 438 (50.8) | 431 (40.2) | 0.678 |
| **Leucocytes** (mm^3^) | 11.700 (4,900) | 8.700 (3,700) | 0.093 |
| **Hemoglobin** (g/dL) | 11.5 (1.3) | 10.6 (1.5) | 0.452 |
| **Aspartate transaminase** (mg/dL) | 35.2 (13.5) | 34.0 (15.1) | 0.096 |
| **Alanine transaminase** (mg/dL) | 32.2 (16.4) | 29.2 (19.9) | 0.188 |
| **Creatine phosphokinase** (IU/L) | 187.8 (59.4) | 179.2 (89.4) | 0.852 |
| **Fibrinogen** (mg/dL) | 134.0 (64.2) | 128.4 (54.3) | 0.956 |
| **Platelets** (mm^3^) | 249,500 (72,400) | 235,300 (75,200) | 0.723 |
| **International Normalized Ratio** | 0.9 (0.3) | 0.8 (0.2) | 0.255 |
| **Erythrocyte sedimentation rate** (mm/hour) | 14.6 (10.1) | 15.6 (10.9) | 0.146 |

**Legend:** Group A is the freeze-dried trivalent antivenom (FDTAV); Group B is available *Bothrops, Bothrops-Lachesis* and *Bothrops-Crotalus* AVs provided by the MoH (SLAV).

**Reference values: Urea** (High: >40 mg/dL), **Creatinine** (High: >1.2 mg/dL), **K^+^** (Low: <3.6 mmol/L), **Na^2+^** (High: >145 mmol/L), **Lactate dehydrogenase** (High: >423 mg/dL), **Leucocytes** (High: >10,000 mm^3^), **Hemoglobin** (Low (female: <12 g/dL; male: <13 g/dL), **Aspartate transaminase** (High: >38 mg/dL), **Alanine transaminase** (High: >44 mg/dL), **Creatine phosphokinase** (High: >190 U/L), **Fibrinogen** (Low: <150 mg/dL), **Platelets** (Low: <150.000 mm^3^), **International Normalized Ratio** (High: >1), **Erythrocyte sedimentation rate** (High: >6 mm in the first hour).
