# Supplementary material for: Safety and efficacy of a freeze-dried trivalent antivenom for snakebites in the Brazilian Amazon: An open randomized controlled phase IIb clinical trial
Source: PLoS Negl Trop Dis. 2017 Nov 27;11(11):e0006068. doi: 10.1371/journal.pntd.0006068 (PMC5720814; doi:10.1371/journal.pntd.0006068)
Supplement: S3 File — For Lachesis snakebites, fibrinogen, clotting time and INR presented normal values 24 hours after AV therapy, for freeze-dried trivalent antivenom (FDTAV) and Ministry of Health standard liquid antivenoms (SLAV) treated groups. Creatinine levels were normal since the admission. (PPTX) [file pntd.0006068.s007.pptx]

## Slide 1
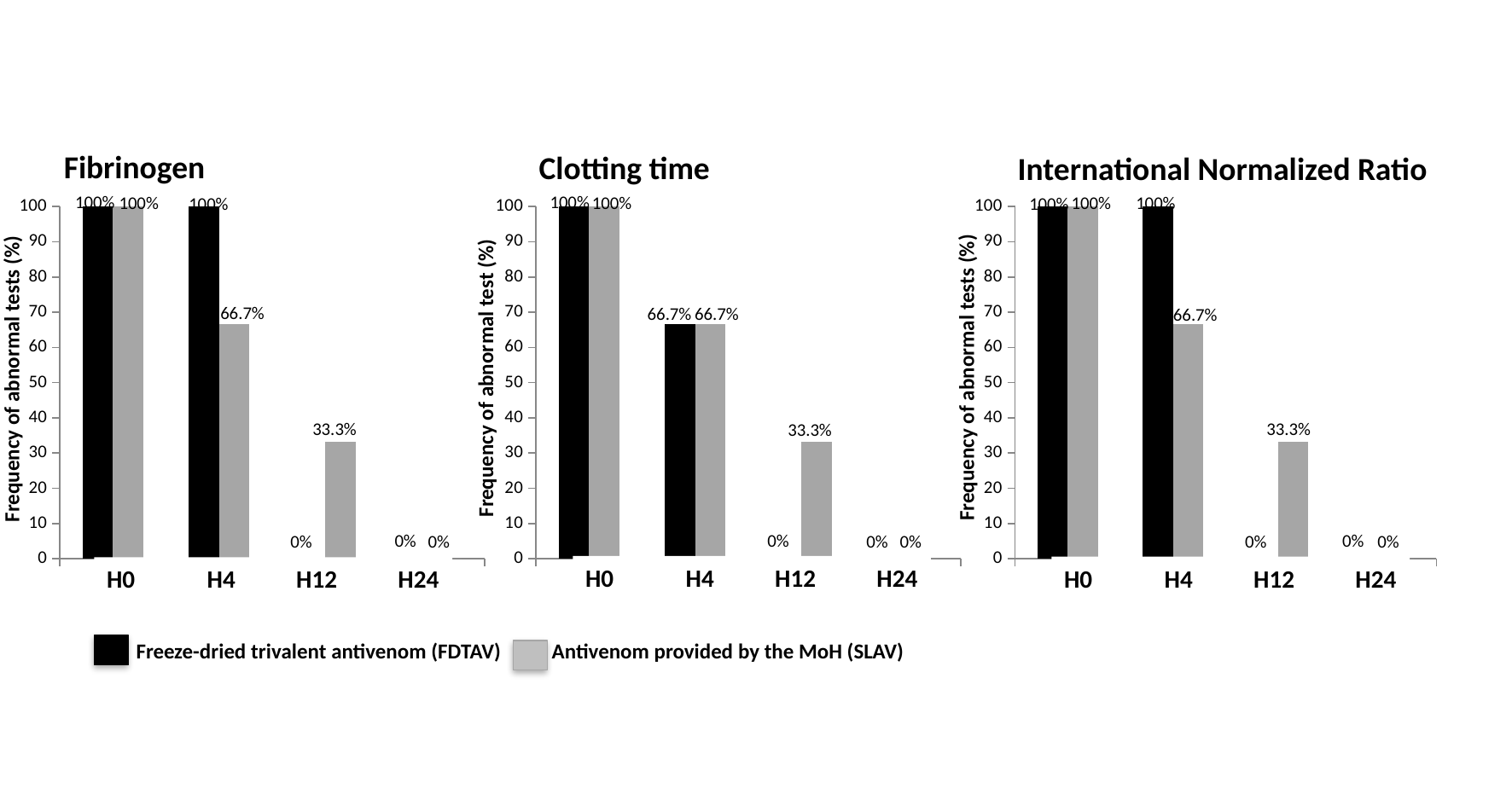

Fibrinogen
Clotting time
International Normalized Ratio
100%
100%
100%
100%
100%
100%
100%
100%
### Chart
| Category | | |
|---|---|---|
### Chart
| Category | | |
|---|---|---|
### Chart
| Category | | |
|---|---|---|66.7%
66.7%
66.7%
66.7%
Frequency of abnormal tests (%)
Frequency of abnormal test (%)
Frequency of abnormal tests (%)
33.3%
33.3%
33.3%
0%
0%
0%
0%
0%
0%
0%
0%
0%
H0 H4 H12 H24
H0 H4 H12 H24
H0 H4 H12 H24
Freeze-dried trivalent antivenom (FDTAV)
Antivenom provided by the MoH (SLAV)
