# Supplementary material for: Safety and efficacy of a freeze-dried trivalent antivenom for snakebites in the Brazilian Amazon: An open randomized controlled phase IIb clinical trial
Source: PLoS Negl Trop Dis. 2017 Nov 27;11(11):e0006068. doi: 10.1371/journal.pntd.0006068 (PMC5720814; doi:10.1371/journal.pntd.0006068)
Supplement: S4 File — For Crotalus snakebites, fibrinogen, clotting time, INR, and creatine phosphokinase presented normal values 24 hours after AV therapy in FDTAV and Ministry of Health standard liquid antivenoms (SLAV) treated groups. (PPTX) [file pntd.0006068.s008.pptx]

## Slide 1
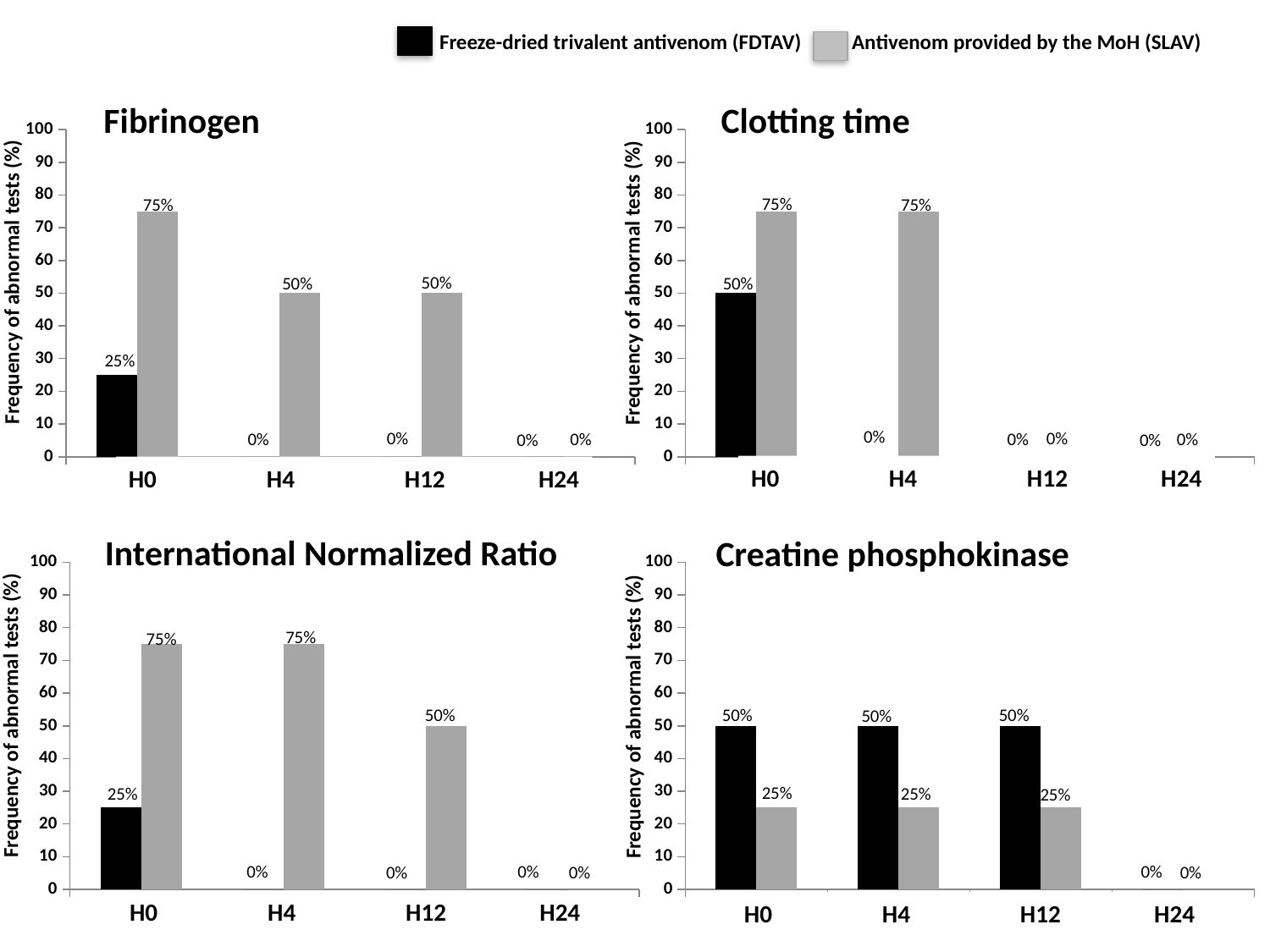

Freeze-dried trivalent antivenom (FDTAV)
Antivenom provided by the MoH (SLAV)
Clotting time
Fibrinogen
### Chart
| Category | | |
|---|---|---|
### Chart
| Category | | |
|---|---|---|75%
75%
75%
Frequency of abnormal tests (%)
Frequency of abnormal tests (%)
50%
50%
50%
25%
0%
0%
0%
0%
0%
0%
0%
0%
0%
H0 H4 H12 H24
H0 H4 H12 H24
International Normalized Ratio
Creatine phosphokinase
### Chart
| Category | | |
|---|---|---|
### Chart
| Category | | |
|---|---|---|75%
75%
Frequency of abnormal tests (%)
50%
Frequency of abnormal tests (%)
50%
50%
50%
25%
25%
25%
25%
0%
0%
0%
0%
 0%
0%
H0 H4 H12 H24
H0 H4 H12 H24
