# Supplementary material for: Safety and efficacy of a freeze-dried trivalent antivenom for snakebites in the Brazilian Amazon: An open randomized controlled phase IIb clinical trial
Source: PLoS Negl Trop Dis. 2017 Nov 27;11(11):e0006068. doi: 10.1371/journal.pntd.0006068 (PMC5720814; doi:10.1371/journal.pntd.0006068)
Supplement: Supplementary file 8 [file pntd.0006068.s003.docx]

Instituições: Instituto de Biologia do Exército – Rio de Janeiro; Instituto Butantan – São Paulo e Fundação de Medicina Tropical do Amazonas.

Introdução: Há quatro gêneros de ofídios com importância médica no Brasil: *Bothrops* , *Crotalus, Lachesis e Micrurus,* sendo o acidente botrópico de maior importância epidemiológica, pois é responsável por cerca de 90% dos acidentes por ofídeos peçonhentos. A soroterapia antiveneno é a única medida específica para o tratamento adequado dos pacientes picados pela maioria dos animais peçonhentos. No Brasil, os imunoderivados são produzidos em formulação líquida, o que requer armazenamento sob temperatura de 2 a 8 graus Celsius positivos Entretanto, a OMS recomenda que os soros sejam produzidos na forma liofilizada. Baseado neste contexto, o Instituto Butantan em parceria com o IBEx iniciou a produção dos primeiros lotes de soro antiofídico liofilizado trivalente (botrópico-laquético-crotálico) que em experimentos subseqüentes demonstrou excelente termoestabilidade em ambientes de elevadas temperaturas, sendo assim dispensada a necessidade de cuidados com refrigeração, tal como ocorre com o soro líquido convencional. Desta forma, torna-se muito útil e prático para aplicação em pontos do território nacional, principalmente na Região Amazônica, atendendo as tropas militares operacionais que realizam manobras, exercícios no terreno, acampamentos, bem como populações indígenas, ribeirinhas e trabalhadores rurais, distantes dos centros de atendimento médico, onde não há sistema de refrigeração.

Objetivos: Avaliar a eficácia da soroterapia realizada com soro antiofídico trivalente liofilizado produzido pelo Instituto Butantan, através de avaliação clínica laboratorial e descrever os possíveis eventos adversos ocorridos até 25 dias após a soroterapia.

População-alvo e Termo de Consentimento Livre e Esclarecido (TCLE): A população-alvo consistirá de indivíduos entre 12 e 70 anos de idade de ambos os sexos, vítimas de ofidismo e com indicação do uso do soro heterólogo após a avaliação pelo médico responsável pelo atendimento inicial. A soroterapia só será iniciada após ciência por parte do voluntário sobre o TCLE concomitantemente com a sua assinatura.Será explicado ao paciente o objetivo do trabalho e sua metodologia, podendo o mesmo questionar qualquer etapa, ou desistir de participar a qualquer momento. Para aplicação de soro liofilizado em crianças entre 12 e 17 anos, deverá ser confeccionado um "termo específico de inclusão de menores" (TCLE especial) e somente após o consentimento do responsável e assinatura do mesmo o tratamento será iniciado.

Metodologia: Será realizado um ensaio clínico em vítimas de ofidismo (voluntários). A captação de voluntários e o acompanhamento clínico-laboratorial serão realizados na região amazônica por profissionais de saúde previamente treinados. O material para coleta de dados e acompanhamento será distribuído no período de treinamento. A captação dos voluntários será realizada no atendimento inicial do acidentado. Esse estudo visa ainda comparar o soro liofilizado com o líquido em utilização rotineira. Para tal, será realizado um sorteio, que definirá o tipo de soro que será administrado no paciente. Caso seja sorteado o soro liofilizado, o médico irá explicar a importância e os objetivos da pesquisa. O paciente que aceitar participar da pesquisa receberá o TCLE assim como os responsáveis por crianças entre 12 e 17 anos receberão o TCLE especial. Após o concorde será iniciado o protocolo de pesquisa que consiste de entrevista e preenchimento de formulários com história do paciente (pregressa e acompanhamento), realização de exames e aplicação do soro liofilizado.

Parecer do CEP: Após discussão, análise e esclarecimento em reuniões sobre as alterações relativas às Instituições envolvidas no projeto, deliberou-se pela **APROVAÇÃO** em 18 de Setembro de 2003

ROBERTO **ESPEDITO DOS SANTOS** RODRIGUES-
Cel Coordenador do CEP/IBEx
